# Mettl14 mediates the inflammatory response of macrophages in atherosclerosis through the NF-κB/IL-6 signaling pathway

**DOI:** 10.1007/s00018-022-04331-0

**Published:** 2022-05-22

**Authors:** Yang Zheng, Yunqi Li, Xianwen Ran, Di Wang, Xianghui Zheng, Maomao Zhang, Bo Yu, Yong Sun, Jian Wu

**Affiliations:** 1 Department of Cardiology, The Second Affiliated Hospital of Harbin Medical University, Harbin, China; 2 The Key Laboratory of Myocardial Ischemia, Chinese Ministry of Education, Harbin, China

**Keywords:** Atherosclerosis, m^6^A modification, Mettl14, Macrophage, Inflammatory response

## Abstract

The inflammatory response of macrophages has been reported to play a critical role in atherosclerosis. The inflammatory state of macrophages is modified by epigenetic reprogramming. m^6^A RNA methylation is an epigenetic modification of RNAs. However, little is known about the potential roles and underlying mechanisms of m^6^A modification in macrophage inflammation. Herein, we showed that the expression of the m^6^A modification “writer” Mettl14 was increased in coronary heart disease and LPS-stimulated THP-1 cells. Knockdown of Mettl14 promoted M2 polarization of macrophages, inhibited foam cell formation and decreased migration. Mechanistically, the expression of Myd88 and IL-6 was decreased in Mettl14 knockdown cells. Through m^6^A modification, Mettl14 regulated the stability of Myd88 mRNA. Furthermore, Myd88 affected the transcription of IL-6 via the distribution of p65 in nuclei rather than directly regulating the expression of IL-6 through m^6^A modification. In vivo, Mettl14 gene knockout significantly reduced the inflammatory response of macrophages and the development of atherosclerotic plaques. Taken together, our data demonstrate that Mettl14 plays a vital role in macrophage inflammation in atherosclerosis via the NF-κB/IL-6 signaling pathway, suggesting that Mettl14 may be a promising therapeutic target for the clinical treatment of atherosclerosis.

**Supplementary Information:**

The online version contains supplementary material available at 10.1007/s00018-022-04331-0.

## Introduction

Monocytes and macrophages are regarded as the main actors in atherosclerosis because of their ubiquitous involvement during all stages of atherosclerosis [4, 9]. Considerable amounts of evidence support that atherosclerosis is a chronic inflammatory disease starting with the entry of monocytes from peripheral blood into the endothelium: rolling, adhesion, activation and migration. Then, monocytes differentiate into macrophages [5, 31]. Depending on the markers, production of specific factors and biological functions of the macrophages, macrophages can be divided into two major subtypes: classically activated macrophages (M1) and alternatively activated macrophages (M2). High levels of the glycoprotein Ly6C (Ly6C^high^) monocytes in mice, known as CD14 (CD14^++^CD16^−^) in humans, differentiate into M1 macrophages. However, Ly6C^low^ monocytes (CD14^+/low^CD16^+^ in humans) differentiate into M2 macrophages [15, 43]. M1 macrophages initiate and sustain inflammation, producing inflammatory cytokines (TNF-α, IL-1β and IL-6) and leading to foam cell formation. M2 macrophages counteract inflammation and secrete anti-inflammatory cytokines (IL-10 and TGF-β) [5, 29]. The biological characteristics of macrophages in atherosclerotic plaques determine the size, composition and stability of the lesion [25]. Recent studies suggest that a substantial proportion of patients retain the progression of the plaque despite achieving guideline-directed therapeutic targets caused by inflammation [36, 40]. Regulating the inflammatory state and function of macrophages raises hope for atherosclerosis regression.

Macrophages are associated with epigenetic reprogramming and are modified by epigenetic enzymes [23]. N^6^-methyladenosine (m^6^A) RNA methylation is a posttranscriptional epigenetic modification of eukaryotic RNAs, including coding and noncoding RNAs [10]. The m^6^A modification process is jointly regulated by three types of enzymes: “writers” (methyltransferases), “erasers” (demethylases) and “readers” (m^6^A-related binding proteins) [41]. Previous research has established that m^6^A modification is associated with RNA metabolism, including changes in RNA structure, splicing, export and translation [19]. Numerous studies have demonstrated that regulators of m^6^A methylation are involved in multiple physiological and pathological processes, such as immune system development, human cancers and cardiovascular disease [19].

However, our understanding of the regulation of m^6^A modification in atherosclerosis is still in its infancy. Guo et al. showed that IFN regulatory factor-1 induced macrophage pyroptosis by Mettl3-mediated m^6^A modification of circ_0029589 in atherosclerosis [16]. Zhang et al. demonstrated that Mettl14 increased the m^6^A modification of pri-miR-19a to promote endothelial cell proliferation and invasion in vitro [49]. Similar studies have reported that Mettl14 regulates endothelial inflammation and that Mettl14 knockdown reduces the development of atherosclerotic plaques [18]. However, the amount of published data on the m^6^A modification of macrophages in atherosclerosis is limited.

Here, we found that the Mettl14 “writer” can regulate the inflammatory state of macrophages in atherosclerosis. Knockdown of Mettl14 promoted M2 polarization and inhibited foam cell formation. Mechanistically, RNA sequencing (RNA-seq) analysis revealed that Mettl14 regulated the expression of Myd88 through m^6^A modification. Myd88 affected the transcription of IL-6 through p65 by regulating the distribution of p65 in nuclei. In vivo, Mettl14 gene knockout in mice significantly reduced the development of atherosclerosis by decreasing the inflammatory response of macrophages. Collectively, our study offers important insights into the m^6^A modification in atherosclerosis and highlights a potential target for treatment.

## Materials and methods

### Study population and ethics statement

A total of 54 participants were selected for our study from the Second Affiliated Hospital of Harbin Medical University between December 2020 and June 2021: 9 controls, 18 patients with ST-segment elevation myocardial infarction (STEMI), 11 patients with non-ST-segment elevation myocardial infarction (NSTEMI) and 17 patients with unstable angina (UA). Inclusion and exclusion criteria were previously described in detail [26]. Briefly, patients with UA, STEMI and NSTEMI were enrolled according to the guidelines [44]. All participants or their families provided informed consent for inclusion before participation in the study, conforming to the Declaration of Helsinki. The current study was approved by the Ethics Committee of the Second Affiliated Hospital of Harbin Medical University, China (KY2020-156).

### Animal and atherosclerosis induction

Mettl14 heterozygous mice (Mettl14^−/+^) were established from C57/BL6 mice by Cyagen Biosciences, Inc. (Suzhou, Jiangsu, China), using CRISPR/Cas9-based targeting and homology-directed repair. C57/BL6 and APOE^−/−^ mice were purchased from Beijing Vital River Laboratory Animal Technology (Beijing, China). Mettl14^−/+^APOE^−/−^ mice were generated by breeding Mettl14^−/+^ mice with APOE^−/−^ mice. Eight- to 10-week-old male APOE^−/−^ (WT) mice and Mettl14^−/+^APOE^−/−^ (KO) mice were fed a high-cholesterol diet (D12108C, Opensource diets) for 12 weeks. Then, the mice were euthanized for further analysis. All mice were housed under specific pathogen-free (SPF) conditions with controlled temperature and a 12-h light/dark cycle at the Second Affiliated Hospital of Harbin Medical University. All experimental protocols were approved by the Institutional Animal Care and Use Committee at the Second Affiliated Hospital of Harbin Medical University (sydwgzr2020-095). This study was conducted in accordance with the Guide for the Care of Use of Laboratory Animals (Institute of Laboratory Animal Resources/National Institutes of Health, Bethesda, MD, USA).

### Peripheral blood mononuclear cells isolated

Overnight fasting blood samples were collected by venipuncture from all participants when they were hospitalized on the first day. The peripheral blood of mice was collected when the mice were fed a high-cholesterol diet for 12 weeks. Peripheral blood mononuclear cells (PBMCs) of humans and mice were isolated using the density gradient technique (TBD; Tianjin, China). Human PBMCs were lysed with TRIzol reagent (Invitrogen, Carlsbad, USA) for RNA extraction. The PBMCs of mice were further analyzed via flow cytometry.

### RNA extraction and qRT-PCR

Total RNA from PBMCs of patients and THP-1 cells was extracted using TRIzol reagent (Invitrogen, Carlsbad, USA) and converted to complementary DNA by a Transcriptor First Strand cDNA Synthesis Kit (Roche, Basel, Switzerland). qRT-PCR was performed with a Transcriptor First Strand cDNA Synthesis Kit (Roche, Basel, Switzerland). The settings were as follows: 40 cycles of 10 s at 95 °C, 30 s at 60 °C, and 30 s at 72 °C. All relative mRNA expression levels were analyzed using the 2^−ΔΔCt^ method. The primers used are listed in Table S1.

### Dot blot assays

Total RNA from PBMCs of patients and THP-1 cells was extracted using TRIzol reagent (Invitrogen, Carlsbad, USA). The RNAs (200, 100 and 50 ng) were double diluted, denatured by heating at 95 °C for 5 min and chilled on ice immediately. The RNAs were then spotted onto nitrocellulose membranes (Solarbio, Beijing, China). Then, the membranes were ultraviolet (UV) crosslinked, blocked and incubated with an m6A-specific antibody (Synaptic Systems, Gottingen, Germany). The other membrane was stained with methylene blue as a loading control.

### Culture and transfection of THP-1 cells

The human macrophage cell line THP-1 was purchased from the American Type Culture Collection. The cells were cultured in RPMI 1640 medium (Gibco, Thermo Fisher Scientific, Waltham, MA, USA) consisting of 10% FBS (Biological Industries, Israel) at 37 °C with 5% CO_2_. THP-1 cells were seeded in 6-well plates (1 × 10^6^ cells/ml) and cultured with 100 ng/ml PMA (Sigma-Aldrich, St. Louis, MO, USA) for 48 h. The cells were treated with TNF-α (10 ng/ml, PeproTech, Cranbury, NJ, USA), LPS (500 ng/ml; Sigma-Aldrich) or IL-4 (20 ng/ml; PeproTech) for 24 h.

The siRNAs for siMettl14 and siMyd88 and the plasmid DNA were purchased from GenePharma (Shanghai, China). All the sequences of the siRNAs are provided in Table S1. The siRNAs were transfected into cells by Lipofectamine RNAimax (Invitrogen, Thermo Fisher Scientific, Waltham, MA, USA) for 48 h. The plasmid DNAs were then transfected into cells by Lipofectamine 3000 (Invitrogen, Thermo Fisher Scientific, Waltham, MA, USA) for 48 h. Then, the media were removed and replaced with fresh media. The cells treated with LPS (500 ng/ml; Sigma-Aldrich) for 24 h.

### Establishment of endotoxemia mice

Mice were intraperitoneally administered LPS (20 mg/kg; Sigma-Aldrich, St. Louis, MO, USA) dissolved in PBS. Control mice were injected PBS. Peritoneal macrophages were isolated 24 h after LPS injection.

### Isolation of bone marrow-derived macrophages (BMDMs)

BMDMs were isolated using standard protocols [7, 28]. In brief, bone marrow cells were flushed out from the femurs and tibias of 8-week-old male APOE^−/−^ and Mettl14^−/+^APOE^−/−^ mice. Primary macrophages were cultured for 7 days in RPMI-1640 medium (Gibco, Thermo Fisher Scientific, Waltham, MA, USA) containing macrophage colony-stimulating factor (M-CSF, 10 ng/ml; PeproTech) and then treated with LPS (200 ng/ml; Sigma-Aldrich) on day 7 for 24 h for further analysis.

### Western blot and antibodies

Proteins were isolated from THP-1 cells in RIPA buffer (Beyotime, Shanghai, China) together with a 1% cocktail of protease (Roche, Basel, Switzerland) and 1 mM PMSF (Beyotime, Shanghai, China). Protein quantification was performed using a BCA protein detection kit (Beyotime, Shanghai, China). Protein lysates were separated by 8–12.5% SDS-PAGE and transferred onto 0.22 μm Immobilon-NC gels (Merck Millipore, Billerica, MA, USA). The primary antibodies included anti-Mettl14, anti-p65, anti-p-p65, anti-Iκbα, anti-ABCA1, anti-ABCG1, anti-PPAR-γ, anti-LXR-α, anti-Myd88, anti-IL-6, anti-β-actin, and anti-GAPDH. Nuclear and cytoplasmic proteins were extracted using a nuclear and cytoplasmic protein extraction kit (Beyotime). The membranes were blocked in blocking buffer and then incubated with the primary antibodies overnight. The protein bands were visualized using chemiluminescence in a Tanon 5100 system (Tanon, Shanghai, China).

### Flow cytometry analysis

THP-1 cells were treated with LPS (500 ng/ml) after transfection. The positive control groups for M1 and M2 were treated with LPS (500 ng/ml) or IL-4 (20 ng/ml), respectively. The cells were harvested and stained with PE-CD86 and APC-CD163 antibodies (BioLegend, San Diego, CA, USA) for 30 min at 4 °C in the dark. Then, the cells were washed, fixed and permeabilized using Fix/Perm buffer (Invitrogen, Thermo Fisher Scientific, Waltham, MA, USA). FITC-CD68 (BioLegend, San Diego, CA, USA) was added to the cells for 30 min at room temperature.

BMDMs were stimulated with LPS (200 ng/ml) or IL-4 (20 ng/ml) on day 7, harvested after stimulation with LPS for 24 h, and then stained with PE-CD86 and FITC-F4/80 antibodies (BioLegend, San Diego, CA, USA) for 30 min at 4 °C in the dark to detect M1 macrophages. To detect M2 macrophages, the BMDMs were stained with the PE-F4/80 antibody (BioLegend) for 30 min at 4 °C in the dark. The cells were then washed, fixed and permeabilized using the Fix/Perm buffer (Invitrogen, Thermo Fisher Scientific, Waltham, MA, USA), followed by incubation with an APC-CD206 antibody (Invitrogen, Thermo Fisher Scientific) for 30 min at room temperature.

The PMBCs of mice were stained with FITC-Ly-6c and PE-CX3CR1 (BioLegend, San Diego, CA, USA) for 30 min at 4 °C in the dark. The data were analyzed using FACSDiva version 6.1.3 (BD Biosciences, San Jose, CA, USA) and FlowJo_V10 (TreeStar, Ashland, OR, USA).

### Intracellular cholesterol assessment

THP-1 cells were treated with 50 μg/ml ox-LDL (YB-002, Yiyuan Biotechnologies, Guangzhou, China) after transfection for 24 h. Then, the cells were washed with PBS and fixed with 4% paraformaldehyde for 20 min. Afterward, the cells were washed with 60% isopropanol and stained with an oil red O staining kit (cultured cells; Solarbio, Beijing, China).

### Wound healing

After stimulation with LPS for 24 h, the cell monolayer was scratched with a sterile pipette tip and washed with PBS. The scratch wound areas were observed at 0 and 24 h.

### Adhesion assay

Endothelial cells were plated in a 96-well plate and grown to full confluence. According to the manufacturer’s instructions, 2 × 10^5^ THP-1 cells stained with 5 µM calcein AM were added to the wells and incubated with the ECs in the 96-well plate for 30 min at 37 °C. The non-adherent cells were removed by rinses with PBS and the numbers of labeled adherent cells were counted under a fluorescence microscope.

### RNA-seq and bioinformatics analysis

RNA from the siM14 and NC groups was isolated using TRIzol reagent. RNA-seq and data analysis were carried out by Sangon Biotech (Shanghai, China). The cDNA library was constructed using a TruSeq PE Cluster Kit v4-cBot-HS (Illumina, USA). Sequencing was performed on a MGISEQ-2000 platform. The RNA-seq reads were mapped by the human reference genome (hg19) from the NCBI using HISAT2. The differentially expressed genes (DEGs) were filtered based on *P* values < 0.05 and fold-changes > 1.5. GO enrichment and KEGG pathway enrichment analyses of DEGs were performed using DAVID Bioinformatics Resources 6.8 (https://david.ncifcrf.gov/).

### Immunofluorescence

The cells were fixed with 4% paraformaldehyde for 20 min. The tissue was cut into 7 μm thin slices and fixed in acetone. Then, the cells or cryosections were immersed in Triton X-100 (Biosharp, Hefei, China) for 30 min followed by supplementation with 10% normal goat serum (Solarbio, Beijing, China) for 30 min. The cells or cryosections were incubated overnight with primary antibodies. Secondary antibodies were added to the cells or cryosections and then labeled with DAPI. The images were captured using a confocal laser scanning microscope (Zeiss LSM 700).

### mRNA decay analysis

THP-1 cells were treated with actinomycin D at a final concentration of 5 μg/ml for 5 or 10 h. Total RNA was extracted at the indicated time points for reverse transcription and qRT-PCR. The mRNA decay rate was normalized to that at 0 h.

### RNA-binding protein immunoprecipitation assays

RIP assays were performed using an RNA Immunoprecipitation Kit (Geneseed Biotech, Guangzhou, China) according to the manufacturer’s instructions. In brief, the cells were harvested in RIP buffer on ice for 10 min. Then, 100 μL RIP lysis was used as input. Then, the cell lysates were incubated with 5 μg anti-m^6^A antibody or control IgG containing protein A/G-agarose beads. After rotation at 4 °C for 2 h, the beads were washed. The immunoprecipitated and input RNAs were isolated and subjected to RIP-qPCR analysis. The PCR primers used are listed in Table S1.

### Chromatin immunoprecipitation (CHIP) assays

ChIP assays were performed using a SimpleChIP Enzymatic Chromatin IP Kit (Cell Signaling Technology, Danvers, MA, USA). The cells were crosslinked with 1% formaldehyde for 10 min and then sonicated in lysis buffer. Ten microliters of the lysate was used as an input. The remaining lysate was subjected to a ChIP assay using p65 or IgG antibodies. The PCR primers used are provided in Table S1.

### Pathological staining

The hearts from the mice were perfused with PBS and immediately embedded in Tissue OCT-Freeze Medium (Tissue-Tek, Sakura, Torrance, CA). Serial cryosections (7 μm) were made using a cryostat. The serial cryosections from the caudal aortic sinus to the proximal aorta were stained with hematoxylin and eosin (HE) to visualize the plaques, oil red O to evaluate the lipid content, and Masson trichrome to analyze lesion size, collagen content, fibrous cap thickness and necrotic core formation. The HE staining kit, oil red O staining kit and Masson trichrome staining kit were purchased from Solarbio (Beijing, China).

### ELISA analysis

Plasma was collected from the mice. Then, the levels of IL-6 were assessed with ELISA kits (Lianke Biotech, Hangzhou, China) according to the manufacturer’s instructions. The absorbance was measured using a microplate reader (Tecan Infinite M200).

### Serum lipid determination

Sera were collected from the mice. Serum lipids, including triglycerides, total cholesterol, LDLs and HDLs, were determined based on commercial kits (Jiancheng, Nanjing, China) using a microplate reader (Tecan Infinite M200).

### Statistical analysis

The results are presented as the means ± SDs. Statistical analyses were performed using the GraphPad Prism version 9.1.0 or SPSS 23.0 software (IBM). Two-group comparisons were performed by unpaired two-tailed Student’s t test, and three or more group comparisons were performed by ordinary one-way analysis of variance (ANOVA). *P* values < 0.05 were considered statistically significant. All experiments were performed at least three times.

## Results

### Mettl14 is upregulated in patients with coronary heart disease and inflammatory macrophages

**Fig. 1:**
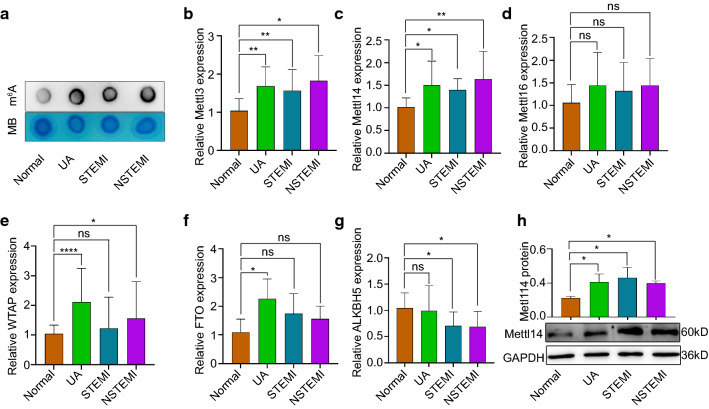
Expression of m^6^A modification methyltransferases and demethylases in coronary heart disease. **a** The m^6^A levels of PBMC RNA in CHD diseases, including UA, STEMI, and NSTEMI. mRNA levels of Mettl3 (**b**), Mettl14 (**c**), Mettl16 (**d**), WTAP (**e**), FTO (**f**), and ALKBH5 (**g**) in UA, STEMI and NSTEMI patients. Control = 9 volunteers, UA = 17 patients, STEMI = 18 patients, NSTEMI = 11 patients. The data are expressed as the mean ± SDs. *P* values were determined by one-way ANOVA with Fisher’s least significant difference (LSD) post hoc test. *P < 0.05; **P < 0.01; ****P < 0.0001; ns, not significant. **h** Protein level of Mettl14 in PBMCs from patients with coronary heart disease. *n* = 5 per group. The data are expressed as the mean ± SD. *P* values were determined by one-way ANOVA with Fisher’s LSD post hoc test. *P < 0.05. MB, methylene blue. PBMC, peripheral blood mononuclear cell. UA, unstable angina. STEMI, ST-segment elevation myocardial infarction. NSTEMI, non-ST-segment elevation myocardial infarction

**Fig. 2:**
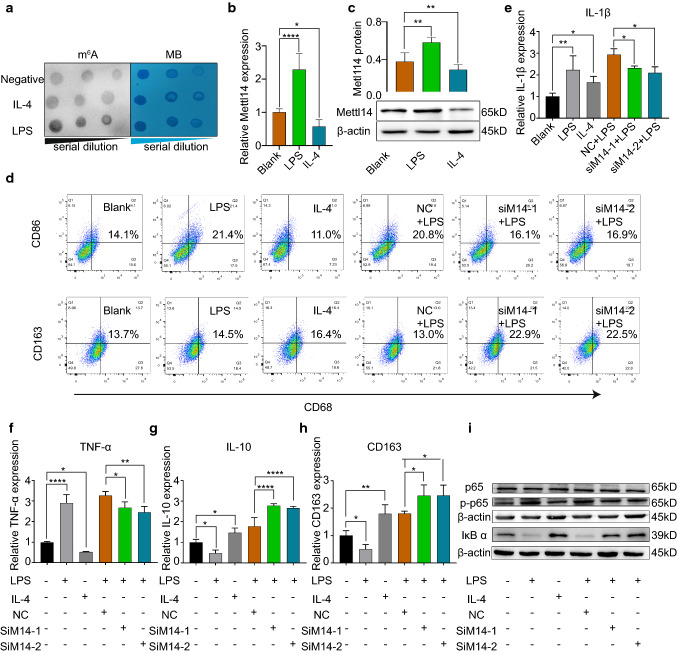
Knockdown of Mettl14 promotes M2 polarization of macrophages. **a** Dot blot assay using an anti-m^6^A antibody in THP-1 cells stimulated with IL-4 (20 ng/ml) or LPS (500 ng/ml). **b** Mettl14 mRNA was measured by qRT-PCR in THP-1 cells stimulated with IL-4 (20 ng/ml) or LPS (500 ng/ml). *n* = 5 per group. The data are expressed as the means ± SDs. *P* values were determined by one-way ANOVA with Fisher’s LSD post hoc test. *P < 0.05; ****P < 0.0001. **c** Mettl14 protein was detected by western blotting. *n* = 3 per group. The data are expressed as the mean ± SD. *P* values were determined by one-way ANOVA with Fisher’s LSD post hoc test. **P < 0.01. **d–i** THP-1 cells treated with NC, siM14-1, or siM14-2 before treatment with LPS (500 ng/ml) or IL-4 (20 ng/ml). **d** The percentage of M1 polarization (CD68^+^CD86^+^) and M2 polarization (CD68^+^CD163^+^) was detected by flow cytometry. The mRNA levels of IL-1β (**e**), TNF-α (**f**), IL-10 (**g**) and CD163 (**h**) were analyzed by qRT-PCR. *n* = 3 per group. The data are expressed as the means ± SDs. *P* values were determined by one-way ANOVA with Fisher’s LSD post hoc test. *P < 0.05; **P < 0.01; ****P < 0.0001. **i** The protein levels of p65, p-p65, and IκB α were detected by western blotting. MB, methylene blue. NC, negative control

The role of m^6^A in PBMCs of patients with coronary heart disease (CHD) was evaluated using an m^6^A mRNA dot blot assay. We divided patients with CHD into the UA group, STEMI group and NSTEMI group. The levels of m^6^A modification, including UA, STEMI and NSTEMI, were significantly increased in CHD (Fig. 1a). Given that the m^6^A modification is primarily catalyzed by methyltransferase (writers) and demethylase (erasers), we hypothesized that the abnormal m^6^A modification in CHD was caused by the dysregulation of m^6^A writers and erasers. Then, we measured the expression of writers (Mettl3, Mettl14, Mettl16 and WTAP) and erasers (FTO and ALKBH5) in the PBMCs of CHD patients. Interestingly, the expression of Mettl3 and Mettl14 was significantly increased in the patients with CHD, including those in the UA, STEMI and NSTEMI groups (Fig. 1b, c, h). The levels of WTAP were increased in the UA and NSTEMI groups but did not change in the STEMI group (Fig. 1e). FTO was upregulated in the UA group but did not change in the STEMI or NSTEMI group (Fig. 1f). The level of ALKBH5 was decreased in the STEMI and NSTEMI groups but did not change in the UA group (Fig. 1g). In addition, Mettl16 did not change significantly in CHD patients (Fig. 1d). Consistent with the results in CHD patients, m^6^A modification in LPS-stimulated THP-1 cells (a human monocyte cell line) increased (Fig. 2a). Moreover, the expression of Mettl3, Mettl14, Mettl16 and WTAP was increased in LPS-stimulated THP-1 cells, and ALKBH5 and FTO expression decreased in LPS-stimulated THP-1 cells (Fig. 2b, c, Fig. S1a-e). In particular, the expression level of Mettl14 was the highest in the LPS-stimulated THP-1 cells. M1 macrophages are polarized by Th1 cytokines (IFN-γ and TNF-α) and pathogen-associated molecular patterns consisting of LPS and lipoproteins [13, 35]. We detected “writers” and “erasers” in THP-1 cells treated with TNF-α. The levels of Mettl3, Mettl14 and Mettl16 were increased in TNF-α-stimulated cells compared with those in the blank control (Fig. S2a–c), while FTO expression was decreased (Fig. S2f). In addition, atherosclerosis is a chronic inflammatory disease that may be caused by endotoxemia and acute inflammation [14]. We established an endotoxemia mouse model via intraperitoneal injection of LPS. Peritoneal macrophages were isolated for qRT-PCR 24 h after the LPS injection. The levels of Mettl3, Mettl14 and WTAP were increased, while the levels of ALKBH5 and FTO were decreased in the LPS group compared with those in the PBS group (Fig. S2g, h, j–l). No change in Mettl16 expression was observed in the LPS group (Fig. S2i). Taken together, these results prompted us to explore the consequences of increased Mettl14 in macrophages caused by atherosclerosis.

### Mettl14 knockdown conditioned macrophages toward M2 polarization and suppressed NF-κB signaling

The m^6^A writer Mettl14 plays a critical role in the regulation of m^6^A modification in macrophages in atherosclerosis. Hence, we knocked down Mettl14 in THP-1 cells through two different siRNAs. qRT-PCR and western blot analysis showed that both siM14-1 and siM14-2 caused a decrease in the expression of Mettl14 (Fig. S3a–c). LPS- and IL-4-stimulated THP-1 cells were used as positive controls for M1 and M2 polarization, respectively (Figs. 2d, S3d, e). As shown in Figs. 2d and S3d, e, knockdown of Mettl14 significantly increased the proportion of M2 macrophages (CD68^+^CD163^+^ cells) and decreased the proportion of M1 macrophages (CD68^+^CD86^+^ cells). Like flow cytometry, qRT-PCR confirmed that the mRNA expression of IL-1β and TNF-α was downregulated and that IL-10 and CD163 were upregulated in siM14-1- and siM14-2-treated THP-1 cells (Fig. 2e–h). To verify our conclusions, we cultured BMDMs from APOE^−/−^ and APOE^−/−^Mettl14^−/+^ mice. The BMDMs were stimulated with LPS (200 ng/ml) for 24 h on the seventh day. qRT-PCR showed decreased levels of the markers of M1 macrophages (TNF-α, Nos2, IL-1β and CCL2) and increased levels of markers of M2 macrophages (Retnlb, CD206 and Arg1) in the APOE^−/−^Mettl14^−/+^ group compared with those in the APOE^−/−^ group (Fig. S4a–g). Flow cytometry confirmed that Mettl14 knockdown promoted macrophage polarization to the M2 phenotype (F4/80^+^CD206^+^ cells) and decreased the proportion of M1 macrophages (F4/80^+^CD86^+^ cells) (Fig. S4h–j). BMDMs undergo polarization to M2 type macrophages after stimulation with IL-4. Next, we compared M1/M2 polarization between the APOE^−/−^ and APOE^−/−^Mettl14^−/+^ groups following IL-4 stimulation. As expected, qRT-PCR revealed decreased expression of the M1 macrophage markers (TNF-α, Nos2, IL-1β and CCL2) and increased expression of the M2 macrophage markers (Retnlb, CD206 and Arg1) (Fig. S4a–g). The percentage of F4/80^+^CD206^+^ cells was increased and the proportion of F4/80^+^CD86^+^ cells was decreased (Fig. S4h–l). IL-4 stimulation also promoted the polarization of Mettl14 knockout BMDMs to M2 macrophages, consistent with the LPS stimulation results. Taken together, the data obtained with primary macrophage cultures confirmed that Mettl14 knockout also promoted macrophage polarization to the M2 phenotype, consistent with the results of the Mettl14 knockdown in THP-1 cells.

Previous studies have demonstrated that the NF-κB pathway plays an important role in macrophage polarization by inducing the expression of inflammatory genes. Then, we investigated the NF-κB pathway using western blotting to evaluate whether the NF-κB pathway was involved in macrophage polarization. Knockdown of Mettl14 markedly reduced the phosphorylation of NF-κB-p65, but the level of p65 was not significantly changed (Figs. 2i, S5a, b, d, e). The expression of IκBα increased in M2 macrophages (Figs. 2i, S5c, f). We added an IκB-α inhibitor (sauchinone) before IL-4 or LPS stimulation of macrophages to confirm whether IκB-α upregulated Mettl14 expression. The inhibition of IκB-α increased the expression of Mettl14, regardless of whether cells were stimulated with IL-4 or LPS (Fig. S5g–i). These results confirmed that IκB-α mediates Mettl14 expression. Taken together, these data suggested that knockdown of Mettl14 prevents the macrophage inflammatory response by promoting M2 polarization via the NF-κB pathway.

### Mettl14 deficiency inhibited foam cell formation and macrophage migration and adhesion

**Fig. 3:**
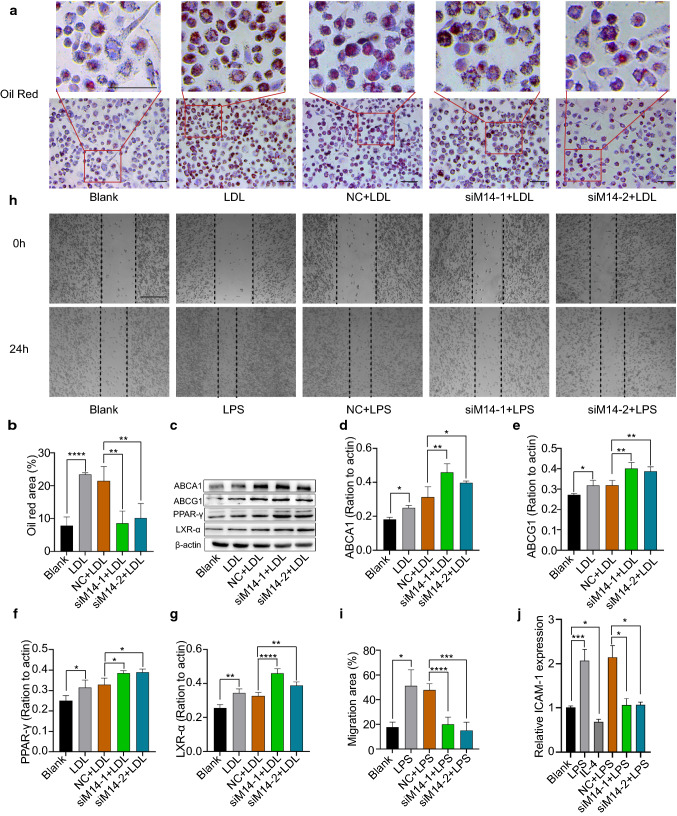
Mettl14 knockdown inhibited foam cell formation and macrophage migration and adhesion. **a**, **b** THP-1 cells were transfected with NC, siM14-1, or siM14-2 before treatment with LDL (50 μg/ml). Foam cell formation was assessed by oil red O staining. Scale bar = 100 μm. *n* = 3 per group. The data are expressed as the means ± SDs. *P* values were determined by one-way ANOVA with Fisher’s LSD post hoc test. **P < 0.01; ****P < 0.0001. **c–g** The ABCA1, ABCG1, PPAR-γ, and LXR-α protein levels were measured by western blotting. *n* = 3 per group. The data are expressed as the means ± SDs. *P* values were determined by one-way ANOVA with Fisher’s LSD post hoc test. *P < 0.05; **P < 0.01; ****P < 0.0001. THP-1 cells were transfected with NC, siM14-1, or siM14-2 before treatment with LPS (500 ng/ml). **h, i** THP-1 cells were transfected with NC, siM14-1, or siM14-2 before treatment with LPS (500 ng/ml). Macrophage migration was detected by wound healing assays. Scale bar = 200 μm. *n* = 3 per group. The data are expressed as the means ± SDs. *P* values were determined by one-way ANOVA with Fisher’s LSD post hoc test. *P < 0.05; ***P < 0.001; ****P < 0.0001. **j** THP-1 cells were transfected with NC, siM14-1, or siM14-2 before treatment with LPS (500 ng/ml). ICAM-1 expression was determined using qRT-PCR. *n* = 3 per group. The data are expressed as the means ± SDs. *P* values were determined by one-way ANOVA with Fisher’s LSD post hoc test. *P < 0.05; ***P < 0.001. NC, negative control

In arteries, foam cell formation and macrophage migration play important roles in the development of atherosclerosis. We asked whether Mettl14 has a role in foam cell formation and macrophage migration. As shown in Fig. 3a, b, the siM14-1 and siM14-2 groups had much less lipid accumulation than the NC group stained with oil red O did. Then, we measured the expression level of lipid metabolism-related proteins. Several studies have shown that ABCA1 and ABCG1 have an essential role in cholesterol efflux during foam cell formation and that PPAR-γ and LXR-α regulate ABCG1/ABCA1 [6]. The expression of ABCA1, ABCG1, PPAR-γ and LXR-α was increased in the Mettl14 knockdown group compared to the NC group (Fig. 3c–g), indicating that the protective function of Mettl14 in macrophages may be related to the PPARγ-LXRα-ABCA1/ABCG1 pathway. Next, using a scratch test, we confirmed that Mettl14 affected migration. The results showed that knockdown of Mettl14 significantly decreased macrophage migration (Fig. 3h–i). The adhesion of endothelial cells to macrophages plays an important role in the development of atherosclerosis. Human umbilical vein endothelial cells were cultured to determine the adhesion ability in different groups of THP-1 cells. As expected, the adhesion ability was reduced in the siMettl14-1 and siMettl14-2 groups compared with that in the NC group (Fig. S6a–f). Meanwhile, ICAM1 expression was significantly decreased in the Mettl14 knockdown group compared with that in the NC group (Fig. 3j). These results showed that the knockdown of Mettl14 inhibited the adhesion of macrophages to endothelial cells. Together, the above findings demonstrated that Mettl14 deficiency inhibited foam cell formation and macrophage migration and adhesion.

### Mettl14 modifies Myd88 mRNA stability by m^6^A modification

**Fig. 4:**
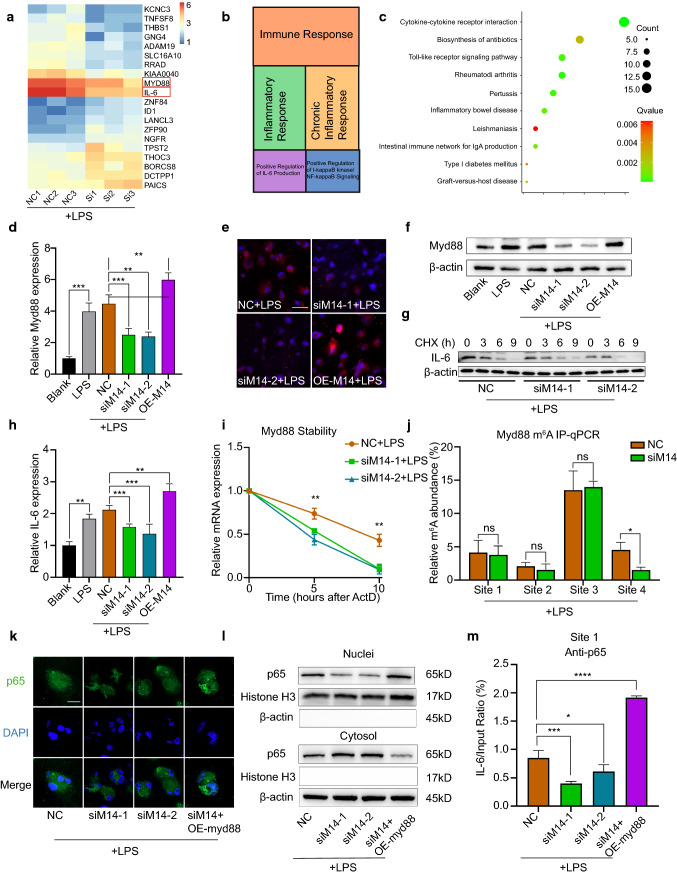
Mettl14 regulates Myd88 via m6A modification and IL-6 through p65. THP-1 cells were treated with NC, siM14-1, siM14-2, overexpressed (OE) Mettl14 or OE Myd88 before treatment with LPS (500 ng/ml). **a** Heat map showing the DEGs in the siMettl14 group and NC group. *n* = 3 per group. **b** Results of a GO analysis performed on the DEGs. **c** Results of a KEGG pathway analysis performed on the DEGs. **d** The expression of Myd88 mRNA was detected by qRT-PCR. *n* = 3 per group. The data are expressed as the means ± SDs. *P* values were determined by one-way ANOVA with Fisher’s LSD post hoc test. **P < 0.01; ***P < 0.001. **e–f** Myd88 protein levels were detected by immunofluorescence (**e**) and western blotting (**f**). Scale bar = 50 μm. **g** At 24 h after transfection, THP-1 cells were treated with cycloheximide (CHX). IL-6 protein was detected by western blotting. **h** IL-6 mRNA levels was measured by qRT-PCR. *n* = 3 per group. The data are expressed as the means ± SDs. *P* values were determined by one-way ANOVA with Fisher’s LSD post hoc test. **P < 0.01; ***P < 0.001. **i** qRT-PCR analysis of Myd88 mRNA stability following treatment with actinomycin D (ActD, 2 μM). *n* = 3 per group. The data are expressed as the means ± SDs. *P* values were determined by one-way ANOVA with Fisher’s LSD post hoc test. **P < 0.01. **j** RIP analysis of the interaction of m^6^A with Myd88 mRNA. *n* = 3 per group. The data are expressed as the means ± SDs, Student’s *t* test. *P < 0.05; ns, not significant. **k** The localization of p65 (green) was observed with immunofluorescence, and the nuclei (blue) were stained with DAPI. Scale bar = 50 μm. **l** The nuclear translocation of p65 was analyzed, and its localization in the cytosol and nucleus was separately quantified using western blotting. **m** ChIP assay results showing the ability of p65 proteins to bind IL-6 promoters. *n* = 3 per group. The data are expressed as the means ± SDs. *P* values were determined by one-way ANOVA with Fisher’s LSD post hoc test. *P < 0.05; ***P < 0.001; ****P < 0.0001

To determine Mettl14-regulated genes in macrophages, we used RNA-seq. For this, we used three samples of Mettl14 knockdown THP-1 cells and three samples of THP-1 cells treated with NC. The RNA-seq data showed that 150 mRNAs were upregulated and that 128 mRNAs were downregulated in the Mettl14 knockdown group compared with the NC group (Fig. 4a, Table S2). Gene Ontology (GO) analysis of these DEGs showed that they were involved mainly in the immune response, inflammatory response, chronic inflammatory response, positive regulation of interleukin-6 production and positive regulation of I-κB kinase/NF-κB signaling (Fig. 4b). The Kyoto Encyclopedia of Genes and Genomes (KEGG) pathway was mainly enriched in cytokine–cytokine receptor interaction, rheumatoid arthritis, pertussis, Toll-like receptor signaling pathway and inflammatory bowel disease (Fig. 4c). These data suggested that Mettl14 regulated the inflammatory response of macrophages.

Among the DEGs, Myd88 and interleukin-6 (IL-6) were very interesting because they play an important role in the inflammatory response of macrophages [1, 3, 8]. First, we detected whether the expression of Myd88 was regulated by Mettl14. qRT-PCR analysis revealed that the mRNA expression levels of Myd88 were markedly lower in Mettl14 knockdown THP-1 cells than in those in the NC group, whereas the mRNA expression of Myd88 was increased in the Mettl14 overexpression group (Fig. 4d). Moreover, using western blots and immunofluorescence staining, we obtained similar results of Myd88 protein expression (Figs. 4e–f, S7a). To determine the regulatory mechanism by which Mettl14 regulates Myd88, we examined the mRNA stability of Myd88. Similar to our hypothesis, the stability of Myd88 mRNA was decreased in the Mettl14 knockdown group (Fig. 4i). This result confirms that Mettl14 may regulate the stability of the Myd88 mRNA through the m^6^A modification. We searched for the 5′-RRACU-3′ sequence in the Myd88 3′-UTR to further confirm that the MyD88 mRNA is a direct target of Mettl14-dependent m^6^A methylation. The sequence analysis revealed four matches for the m6A consensus sequence within the Myd88 3′-UTR (Fig. S8). RNA immunoprecipitation (RIP) analysis revealed that knockdown of Mettl14 reduced the m^6^A modification on site 4 of Myd88 mRNA (Fig. 4j). These results suggested that Mettl14 modifies Myd88 mRNA stability by m^6^A modification.

### Mettl14 regulates the expression of IL-6 by affecting the nuclear distribution of p65 through Myd88

IL-6 is a key inflammatory factor involved in the activation and regulation of macrophages [45]. To validate the RNA-seq results, we performed qRT-PCR to detect the expression of IL-6 mRNA. The level of IL-6 was significantly decreased in the Mettl14 knockdown group, and the expression of IL-6 was the opposite in the Mettl14 overexpression group (Fig. 4h). We detected IL-6 mRNA expression after LPS stimulation at different time points to eliminate the effect of different times of LPS stimulation on IL-6 expression. The highest level of the IL-6 mRNA was detected after 4 h of LPS stimulation, and gradually decreased over time. Comparing the data obtained at each time point, Mettl14 knockdown inhibited IL-6 expression (Fig. S7b). Thus, we postulated that Mettl14 regulates IL-6 expression. Next, we identified the mechanism by which Mettl14 regulates IL-6. Unfortunately, the stability of IL-6 mRNA and translation was not significantly affected by Mettl14 (Figs. 4g, S7c, d). Therefore, Mettl14 does not regulate IL-6 through m^6^A modification. Therefore, how does Mettl14 regulate the expression of IL-6? We assumed that Mettl14 regulated IL-6 through the Myd88/NF-κB pathway according to the prediction of p65 binding to the promoter region of IL-6. Mettl14 did not affect the expression level of p65 (Fig. 2i). Immunofluorescence staining showed that knockdown of Mettl14 impeded p65 translocation from the cytosol to the nucleus (Fig. 4k). However, p65 translocation from the cytosol to the nucleus was facilitated in THP-1 cells treated with siM14 and overexpressing Myd88 (Fig. 4k). To better demonstrate the nuclear and cytoplasmic distribution of p65, we extracted nuclear and cytoplasmic proteins from macrophages. The results showed that siMettl14-1 and siMettl14-2 inhibited the translocation of p65 from the cytoplasm to the nucleus. However, simultaneous overexpression of Myd88 in siMettl14-transfected THP-1 cells reversed the effect of Mettl14 inhibition of p65 translocation to the nucleus (Figs. 4l, S7e, f). As expected, ChIP analysis revealed that knockdown of Mettl14 inhibited p65 binding to the IL-6 promoter (Figs. 4m, S7g). P65 binding to the IL-6 promoter was increased in cotransfected THP-1 cells. These results indicated that Mettl14 regulates its expression through the Myd88/NF-κB pathway.

### Knockdown of Mettl14 induces a low inflammatory response in macrophages through Myd88

**Fig. 5:**
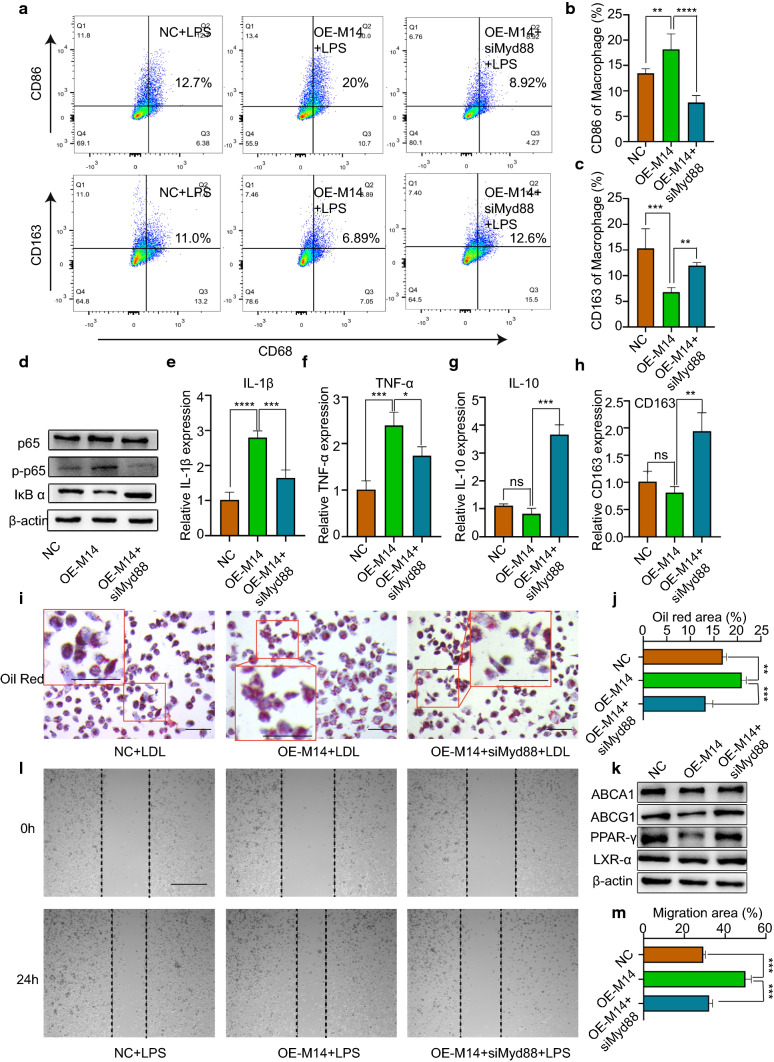
Mettl14 regulates the inflammatory response of macrophages through Myd88. **a–h** and **l–m** THP-1 cells were transfected with NC, OE Mettl14 or OE Mettl14 and siMyd88 before treatment with LPS (500 ng/ml). **a**–**c** The percentage of M1 polarization (CD68^+^CD86^+^) and M2 polarization (CD68^+^CD163^+^) was determined by flow cytometry. *n* = 4 per group. The data are expressed as the means ± SDs. *P* values were determined by one-way ANOVA with Fisher’s LSD post hoc test. **P < 0.01; ***P < 0.001; ****P < 0.0001. **d** The proteins p65, p-p65 and IκB α were measured by western blotting. The mRNA levels of IL-1β (**e**), TNF-α (**f**), IL-10 (**g**) and CD163 (**h**) were analyzed by qRT-PCR. *n* = 3 per group. The data are expressed as the means ± SDs. *P* values were determined by one-way ANOVA with Fisher’s LSD post hoc test. *P < 0.05; **P < 0.01; ***P < 0.001; ****P < 0.0001; ns, not significant. **i–k** THP-1 cells were transfected with NC, siM14-1, or siM14-2 before treatment with LDL (50 μg/ml). **i**, **j** Foam cell formation was detected by oil red O staining. Scale bar = 100 μm. *n* = 3 per group. The data are expressed as the means ± SDs. *P* values were determined by one-way ANOVA with Fisher’s LSD post hoc test. **P < 0.01; ***P < 0.001. **k** The ABCA1, ABCG1, PPAR-γ, and LXR-α protein levels were measured by western blotting. **l**, **m** Macrophage migration was assessed by wound healing assays. Scale bar = 200 μm. *n* = 3 per group. The data are expressed as the means ± SDs. *P* values were determined by one-way ANOVA with Fisher’s LSD post hoc test. ***P < 0.001

We showed that Mettl14 knockdown induced a low inflammatory response in macrophages (Figs. 2, 3) and that Myd88 was regulated by Mettl14 (Fig. 4). We next examined whether the functions of Mettl14 are mediated by Myd88 in macrophages. For this, we conducted rescue experiments. We generated a Mettl14 expression vector, which was cotransfected with siMyd88. Overexpression of Mettl14 promoted M1 polarization rather than M2 polarization in THP-1 cells (Fig. 5a–c). As expected, siMyd88 reversed Mettl14 overexpression-induced M1 polarization (Fig. 5a–c). The expression level of p65 protein did not significantly change in the OE-M14 group or cotransfection group. The p-p65 protein level increased in Mettl14-overexpressing THP-1 cells, and its expression was decreased by siMyd88 (Figs. 5d, S9a–c). We found that the inflammatory factors IL-1β and TNF-α increased and that the anti-inflammatory factors IL-10 and CD163 decreased in Mettl14-overexpressing cells (Fig. 5e–h). Moreover, the levels of inflammatory factors and anti-inflammatory factors were reversed in cells cotransfected with siMyd88. Furthermore, Mettl14 overexpression increased foam cell formation and decreased the abundance of lipid metabolism-related proteins (Fig. 5i–k). However, foam cell formation decreased in response to siMyd88, and lipid metabolism-related proteins increased (Figs. 5i–k, S9d–g). The migration of macrophages had similar results. Overexpression of Mettl14 promoted the migration of macrophages (Fig. 5l–m). However, siMyd88 reversed OE-M14-induced migration. Collectively, these results suggested that Mettl14 regulates the functions of macrophages through Myd88.

### Mettl14 deficiency attenuates atherosclerosis progression in vivo

**Fig. 6:**
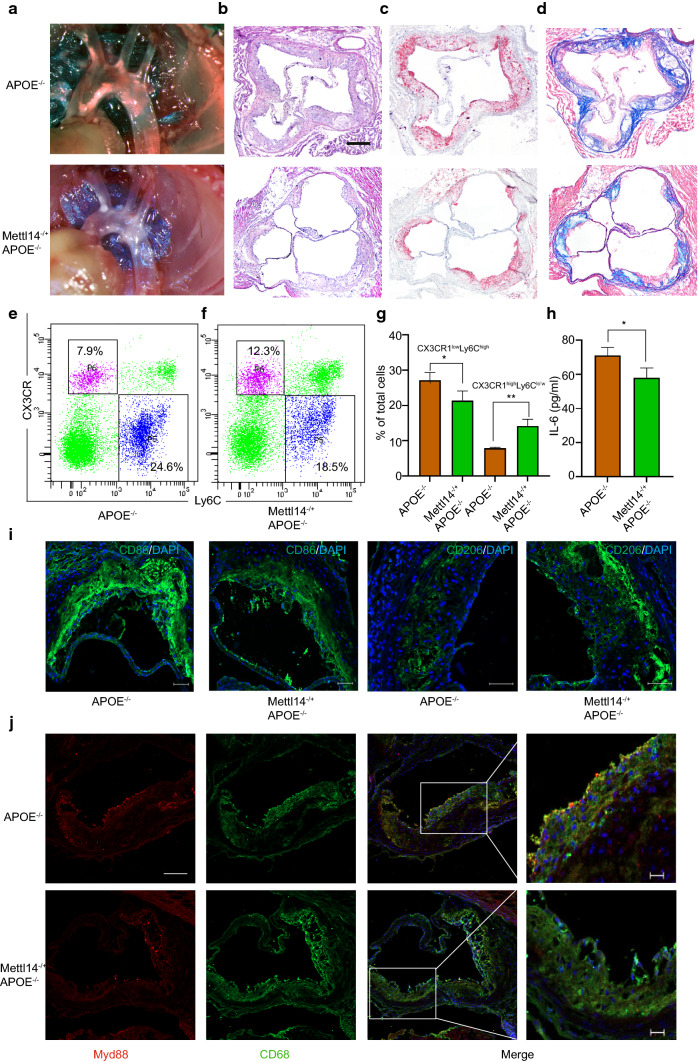
Mettl14 knockout mice undergo the inflammatory response and atherosclerosis development. Mettl14^−/+^APOE^−/−^ (KO) mice and APOE^−/−^ (WT) mice were fed a high-cholesterol diet for 12 weeks. **a** Representative images of aortic arches are shown. Representative images of the aortic root lesion area stained with HE (**b**), oil red O (**c**) and Masson trichrome (**d**). Scale bar = 200 μm. **e–g** Flow cytometry analysis of monocyte populations in the peripheral blood isolated from KO and WT mice. *n* = 3 per group. The data are expressed as the mean ± SD. *P* values were determined by Student’s *t* test. *P < 0.05; **P < 0.01. **h** Levels of IL-6 in the plasma isolated from KO and WT mice. The data are expressed as the mean ± SD. *P* values were determined by Student’s *t*-test. *P < 0.05. **i** Immunofluorescence staining showing the expression of an M1 marker (CD86) and M2 marker (CD206) in atherosclerotic plaques. Scale bar = 50 μm **j** Immunofluorescence staining showing Myd88-positive cells in atherosclerotic plaques. Scale bar = 50 μm. The scale bar of the high power field is 20 μm

Given that Mettl14 regulates the functions of macrophages in vitro, we further assessed the effect of Mettl14 on atherosclerosis progression in vivo. Previous studies have demonstrated that Mettl14 gene knockout causes embryo death. Mettl14 heterozygous knockout mice were used for research [18]. To do this, age- and weight-matched male APOE^−/−^(WT) mice and Mettl14^−/+^APOE^−/−^(KO) mice were fed a western diet for 3 months. After 3 months, the aortas and peripheral blood of these mice were dissected. qRT-PCR and western blotting revealed decreased expression of Mettl14 in BMDMs (Fig. S10a–c). Mettl14 expression was significantly decreased in atherosclerotic plaques of KO mice (Fig. S10d). Compared to the WT mice, the KO mice did not exhibit changes in the plasma lipid profiles (Fig. S10e–h). As shown in Fig. 6a, images of aortic arches suggested that KO mice were significantly protected from atherosclerosis. HE staining of the aortic roots revealed that the lesion areas were smaller in KO mice than in WT mice (Figs. 6b, S10i). The extent of proximal aortic atherosclerosis was reduced in KO mice using oil red O (Figs. 6c, S10j). These results suggested that Mettl14 knockout can significantly reduce atherosclerosis.

Next, using Masson trichrome staining, we examined the characteristics of plaque stabilization between WT and KO mice. Compared to the WT mice, the KO mice had an increased collagen content in the proximal aorta (Figs. 6d, S10k). In addition, the thickness of the fibrous cap was greater in the lesions of KO mice than in those of WT mice (Figs. 6d, S10l). Importantly, the necrotic area was decreased in KO mice vs. WT mice (Figs. 6d, S10m). Taken together, these data showed that Mettl14 deficiency suppressed the characteristics of vulnerable plaque formation.

### Mettl14 regulates the function of macrophages via Myd88/IL-6 in vivo

To verify the function and mechanism of macrophages regulated by Mettl14 in vivo, we analyzed the subtypes of monocytes in the peripheral blood of the mice. The subtypes of monocytes include proinflammatory Ly6C^high^CX3CR1^low^ monocytes and anti-inflammatory Ly6C^low^CX3CR1^high^ monocytes. We found that the subpopulations of Ly6C^high^CX3CR1^low^ monocytes were lower in KO mice than in WT mice and that Ly6C^low^CX3CR1^high^ monocytes were higher (Fig. 6e–g). In plaques of KO mice, Mettl14 expression was significantly decreased compared with that in WT mice (Fig. S10d). The level of the M2 marker CD206 was increased, while that of the M1 marker CD86 was decreased in plaques of KO mice (Fig. 6i). These data indicate that Mettl14 deficiency reprograms monocytes/macrophages to anti-inflammatory effects. Then, we detected the expression of Myd88 in plaque macrophages. Figure 6j shows that the expression of Myd88 significantly decreased in plaque macrophages from KO mice. Surprisingly, we also found that the plasma level of IL-6 was reduced in KO mice (Fig. 6h). In conclusion, the results suggest that Mettl14 can regulate the function of macrophages in atherosclerosis via Myd88/IL-6 in vivo.

## Discussion

The presence of macrophages is a common condition that has considerable impact on all stages of atherosclerosis [4, 9]. The functions of macrophages are mainly regulated by epigenetic reprogramming [23]. Recent evidence suggests that epigenetic modifications can be grouped into three categories: epigenetic triad, DNA methylation, and histone modification and nucleosome positioning [34]. In recent years, the mechanism of epigenetic modifications has been updated with the development of specific methylated RNA immunoprecipitation and next-generation sequencing [32]. M^6^A is the most common epitranscriptomic modification of mRNA from yeast, plants, files, humans and other mammals [50]. M^6^A methylation marks are dynamic and reversible. Briefly, m^6^A methylation occurs at the consensus sequence RRACH (R = G or A; H = A, C or U) through methyltransferases (writers) and demethylases (erasers). Then, the m^6^A-related binding proteins (readers) selectively bind the site of m^6^A modification. After this regulatory event, RNAs are cleaved, stable, degraded and translated [21, 24, 39]. Several studies have demonstrated a link between m^6^A modification and human diseases [39].

Zhang et al. demonstrated that m^6^A modification and Mettl14 were significantly increased in the atherosclerotic vascular endothelial cells of patients with carotid stenosis [49]. Guo et al. showed that the expression of Mettl3 was significantly elevated in macrophages in patients with acute coronary syndrome [16]. Another study showed opposite results, which may be caused by different detection methods for m^6^A modification. The m^6^A levels were significantly decreased in peripheral blood mononuclear cells, as detected by colorimetry. These three studies suggest that m^6^A modification plays an important role in atherosclerosis. Here, we observed that the levels of m^6^A modification and Mettl14 were increased in the peripheral blood mononuclear cells of patients with coronary heart disease and LPS-stimulated THP-1 cells. It is possible, therefore, that Mettl14 has a pivotal role in macrophages in atherosclerosis. Existing studies recognize the critical role played by m^6^A modification in the immune response of macrophages. An earlier study showed that the reader YTHDF2 was upregulated after LPS stimulation and that YTHDF2 knockdown promoted the inflammatory response in LPS-stimulated macrophages [48]. Subsequent studies confirmed that m^6^A modification regulated M1/M2 polarization and cholesterol efflux [27, 33, 46, 47]. However, the function of Mettl14 in macrophages in atherosclerosis has not been reported. In this study, we first reported that Mettl14 regulated the inflammatory state of macrophages in atherosclerosis. Knockdown of Mettl14 in macrophages promotes M2 polarization. Moreover, foam cell formation and migration were inhibited in Mettl14 knockdown macrophages. Atherosclerotic plaques and the inflammatory response were significantly reduced in Mettl14 knockout mice. These data provide evidence that Mettl14 plays an essential role in the regulation of macrophages in atherosclerosis.

The mechanisms of m^6^A modification are diverse, including the fold, stability, degradation and cellular interactions of the modified RNA [32]. Jian et al. demonstrated that Mettl14 enhances the transcription factor FOXO1 by increasing its translation, not RNA stabilization, in endothelial inflammation [18]. In bacterial infection, Mettl14 depletion blocked m^6^A methylation of SOCS1, diminishing its RNA stability [11]. In addition, Mettl14 forms a complex with Mettl3, called the Mettl3/Mettl14 complex, modifying nascent transcripts whose translation is enhanced [17]. We performed RNA-seq of Mettl14-knockdown macrophages to explore the regulatory mechanism of Mettl14. The DEGs regulated by Mettl14 were enriched in the inflammatory response, indicating that Mettl14 plays an important role in the inflammatory response in macrophages. We found two interesting DEGs, those encoding Myd88 and IL-6. qRT-PCR further confirmed that the expression of Myd88 and IL-6 was consistent with the RNA-seq results. Most Toll-like receptors (TLRs) and several cytokine receptors signal through Myd88 to initiate a rapid immune response when alarmins stimulate macrophages [8]. In addition, Myd88 knockout mice showed smaller atherosclerotic plaques and less macrophage activation, lipid accumulation and foam cell formation [3, 30, 42]. Myd88 plays a central role in the inflammatory response of macrophages. Interestingly, Myd88 was found to be a target gene of Mettl14 in our study. Through what mechanism does Mettl14 regulate Myd88? We measured Myd88 mRNA stability in Mettl14 knockdown THP-1 cells. As expected, the stability and m^6^A modification of Myd88 mRNA were decreased in Mettl14 knockdown cells. Thus, Mettl14 regulates Myd88 through m^6^A modification.

IL-6 is a major contributor to the development of atherosclerosis. The aortas of atherosclerotic mice and rats had higher levels of macrophage-attracting IL-6 than did those of the control, and the expression level of IL-6 was higher with aging [2, 12]. The mice were given an anti-mouse IL-6 receptor antibody, and the atherosclerotic lesion size and inflammation were reduced [1]. In clinical trials, the first cytokine inhibition, IL-1β inhibition, was used for atherosclerosis treatment and prevention and achieved results. The results showed that IL-6 was the central signaling cytokine of IL-1β inhibition, in turn reducing the inflammatory response [38]. In a recent RESCUE trial, the IL-6 ligand monoclonal antibody ziltivekimab was highly effective at reducing the inflammatory response and atherosclerotic biomarkers, suggesting that IL-6 has become a new therapeutic target for atherosclerosis [37]. In our study, IL-6 was the target of Mettl14. The expression of IL-6 was decreased in the Mettl14 knockdown, but the stability and translation of IL-6 mRNA did not significantly change in the Mettl14 knockdown. Mettl14 may, therefore, not depend on m^6^A modification to regulate the expression of IL-6. On the basis of our results, we found that Mettl14 regulates the expression of Myd88 via m^6^A modification, which is the upstream regulation of the NF-κB pathway. Several studies have demonstrated that NF-κB/p65 can regulate the transcription of IL-6 [20, 22]. Therefore, we hypothesized that Mettl14 regulates IL-6 through the Myd88/NF-κB pathway. As expected, Mettl14 regulated the distribution of p65 in nuclei, which regulates the transcription of IL-6, reflecting the mechanism of upstream regulation of IL-6 in macrophages.

**Fig. 7:**
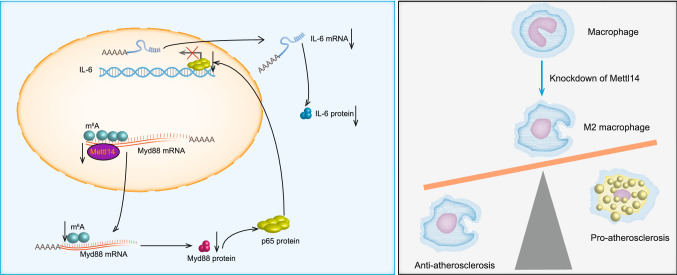
Graphical abstract of Mettl14 regulation of the development of atherosclerosis via the Myd88/IL-6 signaling pathway in macrophages

In summary, our study explores the regulatory mechanisms of macrophage inflammation from a new epigenetic perspective (Fig. 7). Our findings confirmed that Mettl14 can decrease the inflammatory response of macrophages. In vitro, Mettl14 can reduce the stability of Myd88 mRNA via m^6^A modification, thereby increasing M2 macrophage polarization and decreasing foam cell formation and migration of macrophages. Furthermore, we also demonstrated that Mettl14 regulates the transcription of IL-6 through the NF-κB/p65 pathway. In vivo, Mettl14 knockout in mice decreased atherosclerotic plaques and the inflammatory response. Our data indicate that Mettl14 may be a promising therapeutic target of macrophages in the treatment of atherosclerosis.

### Supplementary Information

Below is the link to the electronic supplementary material.

Fig. S1 The expression of “readers” and “writers” in THP-1 cells treated with IL-4 (20 ng/ml) or LPS (500 ng/ml). mRNA expression of Mettl3 (a), Mettl16 (b), WTAP (c), ALKBH5 (d) and FTO (e) was measured by qRT-PCR in THP-1 cells stimulated with IL-4 (20 ng/ml) or LPS (500 ng/ml). n=5 per group. The data are expressed as the mean ± SD. P-values were determined by one-way ANOVA with Fisher’s LSD post-hoc test. *, P<0.05; **, P<0.01; ***, P<0.001; ****, P<0.0001; ns, not significant (PDF 841 KB)

Fig. S2 The expression of “readers” and “writers” in inflammatory macrophages. a-f Expression of the Mettl3 (a), Mettl14 (b), Mettl16 (c), WTAP (d), ALKBH5 (e) and FTO (f) mRNAs was measured in THP-1 cells stimulated with TNF-α (10 ng/ml) using qRT-PCR. n=3 samples per group. The data are presented as the means ± SD. P-values were determined using Student’s t-test. *, P<0.05; ****, P<0.0001; ns, not significant. g-l Expression of the Mettl3 (g), Mettl14 (h), Mettl16 (i), WTAP (j), ALKBH5 (k) and FTO (l) mRNAs was measured in peritoneal macrophages from the mouse endotoxemia model using qRT-PCR. n=4 samples per group. The data are presented as the means ± SD. P-values were determined using Student’s t-test. *, P<0.05; **, P<0.01; ***, P<0.001; ns, not significant (PDF 926 KB)

Fig. S3 Mettl14 knockdown promotes the M2 polarization of macrophages. a-b Expression of the Mettl14 mRNA was measured in THP-1 cells transfected with siMettl14 using qRT-PCR. n=3 per group. The data are expressed as the mean ± SD. P-values were determined by one-way ANOVA with Fisher’s LSD post-hoc test. *, P<0.05; **, P<0.01; ***, P; ****, P<0.0001. c Mettl14 protein level in THP-1 cells transfected with siMettl14. d-e THP-1 cells treated with NC, siM14-1, or siM14-2 before treatment with LPS (500 ng/ml) or IL-4 (20 ng/ml). Percentages of macrophages exhibiting M1 (CD68^+^CD86^+^) and M2 (CD68^+^CD163^+^) polarization were determined using flow cytometry. n=5 per group. The data are expressed as the mean ± SD. P-values were determined by one-way ANOVA with Fisher’s LSD post-hoc test. *, P<0.05; **, P<0.01 (PDF 909 KB)

Fig. S4 Mettl14 knockdown promotes M2 polarization of BMDMs. The BMDMs were stimulated with LPS (200 ng/ml) or IL-4 (20 ng/ml) on day 7 and harvested 24 h later. a-g TNF-α (a), Nos2 (b), IL-1β(c), CCL2 (d), Retnlb (e), CD206 (f), and Arg1 (g) mRNA levels in WT and KO mice. n=4 mice per group. The data are presented as the means ± SD. P-values were determined using Student’s t-test. *, P<0.05; **, P<0.01; ****, P<0.0001. h-l Percentages of macrophages exhibiting M1 (F4/80^+^CD86^+^) and M2 (F4/80^+^CD206^+^) polarization were determined using flow cytometry. n=4 samples per group. The data are presented as the means ± SD. P-values were determined using Student’s t-test. *, P<0.05; **, P<0.01; ***, P<0.001 (PDF 1728 KB)

Fig. S5 Mettl14 knockdown promotes the M2 polarization of macrophages. a-f Protein levels of p65 (a, d), p-p65 (b, e), and IκB-α (c, f) were determined using western blotting. n=3 per group. The data are expressed as the mean ± SD. P-values were determined by one-way ANOVA with Fisher’s LSD post-hoc test. *, P<0.05; **, P<0.01; ***, P<0.001; ns, not significant. g-h Mettl114 protein levels were measured using western blotting. The data are presented as the means ± SD. P-values were determined using Student’s t-test. **, P<0.01. i Mettl14 mRNA expression. The data are presented as the means ± SD. P-values were determined using Student’s t-test. *, P<0.05; ****, P<0.0001 (PDF 1098 KB)

Fig. S6 Mettl14 knockdown inhibited the adhesion of macrophages. a-f THP-1 cells were transfected with NC, OE Mettl14 or OE Mettl14 and siMyd88 before treatment with LPS (500 ng/ml). Representative fluorescence microscopy images of macrophages adhering to endothelial cells are shown (PDF 1761 KB)

Fig. S7 Mettl14 regulates the expression of Myd88 and IL-6. a Myd88 protein levels were determined by western blotting. n=3 per group. The data are expressed as the mean ± SD. P-values were determined by one-way ANOVA with Fisher’s LSD post-hoc test. **, P<0.01; ****, P<0.0001. b IL-6 expression. c At 24 h after transfection, THP-1 cells were treated with cycloheximide (CHX). IL-6 protein levels were determined by western blotting. n=3 per group. The data are expressed as the mean ± SD. P-values were determined by one-way ANOVA with Fisher’s LSD post-hoc test. ns, not significant. d Results of the qRT-PCR-based analysis of IL-6 mRNA stability following treatment with actinomycin D (ActD, 2 μM). n=3 samples per group. The data are presented as the means ± SDs. P-values were determined using one-way ANOVA with Fisher’s LSD post-hoc test. ns, not significant. e-f The nuclear translocation of p65 was analyzed, and its levels in the cytosol and nucleus were quantified separately using western blotting. n=3 per group. The data are presented as the means ± SDs. P-values were determined using one-way ANOVA with Fisher’s LSD post-hoc test. *, P<0.05; **, P<0.01; ***, P<0.001. g Results of the ChIP assay showing the ability of the p65 protein to bind to IL-6 promoters. n=3 per group. The data are expressed as the mean ± SD. P-values were determined by one-way ANOVA with Fisher’s LSD post-hoc test. ***, P<0.001; ns, not significant (PDF 979 KB)

Fig. S8 An analysis of the Myd88 3’-UTR sequence revealed four matches to the 5’-RRACU-3’ m6A consensus sequence (R=A or G) (PDF 144 KB)

Fig. S9 Mettl14 regulates the inflammatory response of macrophages through Myd88. a-c THP-1 cells were transfected with NC, OE Mettl14 or OE Mettl14 and siMyd88 before treatment with LPS (500 ng/ml). d-g THP-1 cells were transfected with NC, siM14-1, or siM14-2 before treatment with LDL (50 μg/ml). Protein levels of p65 (a), p-p65 (b), IκBα (c), ABCA1 (d), ABCG1 (e), PPAR-γ (f) and LXR-α (g) were measured by western blotting. n=3 per group. The data are expressed as the mean ± SD. P-values were determined by one-way ANOVA with Fisher’s LSD post-hoc test. *, P<0.05; **, P<0.01; ***, P<0.001; ns, not significant (PDF 943 KB)

Fig. S10 The development of atherosclerosis is inhibited in Mettl14 knockdown mice. a-c BMDMs were directly harvested on day 7 without stimulation. Mettl14 expression was detected in WT and KO mice using qRT-PCR and western blotting. n=3 animals per group. The data are presented as the means ± SD. P-values were determined using Student’s t-test. *, P<0.05; ***, P<0.001. Levels of triglycerides (e), total cholesterol (f), LDL (g) and HDL (h) in WT and KO mice. n=3 per group. The data are expressed as the mean ± SD. P-values were determined by Student’s t-test. ns, not significant. Quantification of the aortic root lesion area in sections stained with HE (i) and Oil red O (j), along with the collagen area (k), fibrous cap thickness (l) and necrotic core (m). n=3 per group. The data are expressed as the mean ± SD. P-values were determined by Student’s t-test. *, P<0.05; **, P<0.01 (PDF 2858 KB)

Supplementary file11 (DOC 57 KB)

Supplementary file12 (XLS 74 KB)

## Data Availability

Supplementary data to this article can be found online.
