# Supplementary figures and images for: Mettl14 mediates the inflammatory response of macrophages in atherosclerosis through the NF-κB/IL-6 signaling pathway
Source: Cell Mol Life Sci. 2022 May 22;79(6):311. doi: 10.1007/s00018-022-04331-0 (PMC9124663; doi:10.1007/s00018-022-04331-0)

**a**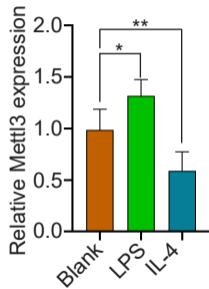**b**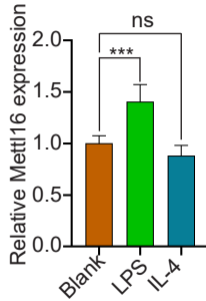**c**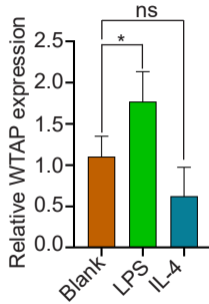**d**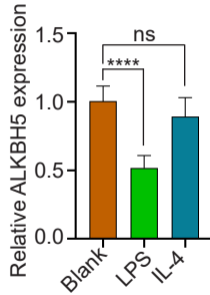**e**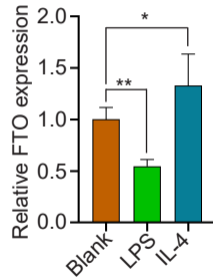

Supplement: Supplementary file 1 — Fig. S1 The expression of “readers” and “writers” in THP-1 cells treated with IL-4 (20 ng/ml) or LPS (500 ng/ml). mRNA expression of Mettl3 (a), Mettl16 (b), WTAP (c), ALKBH5 (d) and FTO (e) was measured by qRT-PCR in THP-1 cells stimulated with IL-4 (20 ng/ml) or LPS (500 ng/ml). n=5 per group. The data are expressed as the mean ± SD. P-values were determined by one-way ANOVA with Fisher’s LSD post-hoc test. *, P<0.05; **, P<0.01; ***, P<0.001; ****, P<0.0001; ns, not significant (PDF 841 KB) [file 18_2022_4331_MOESM1_ESM.pdf]

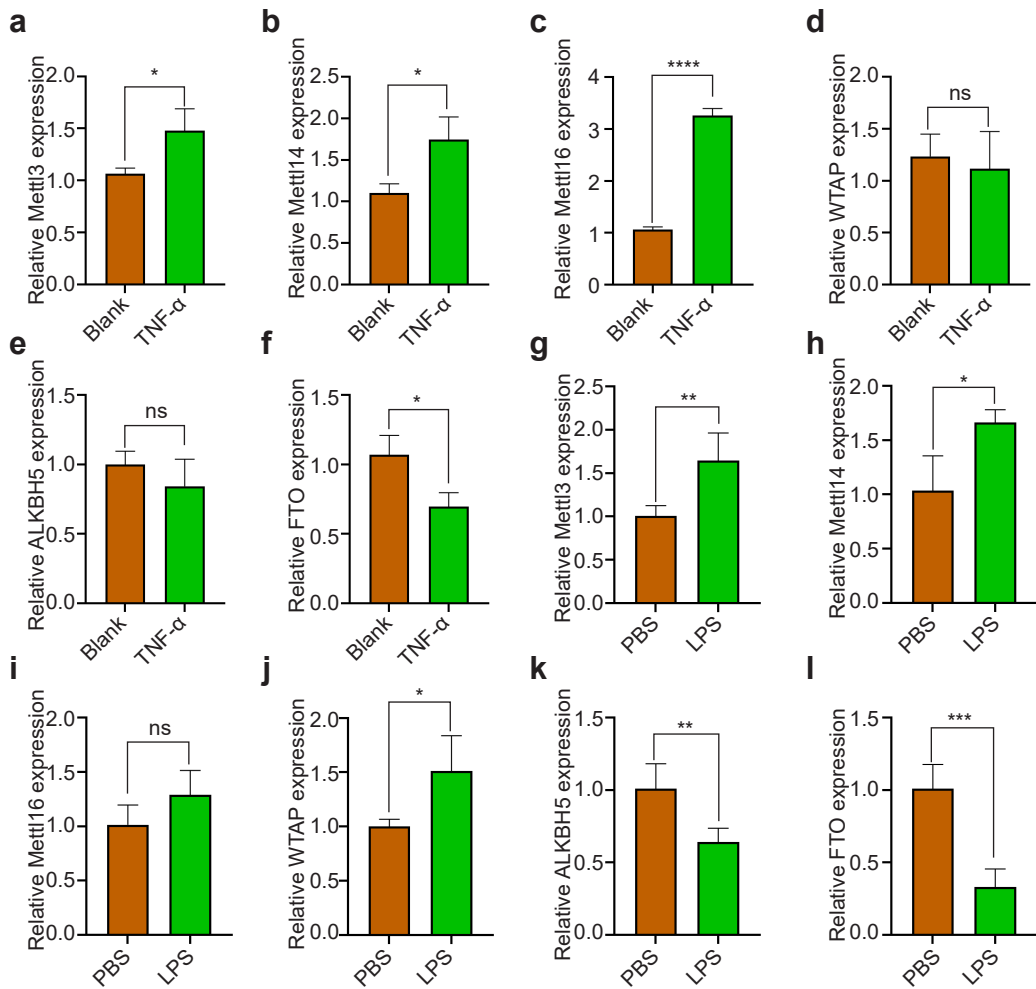

Supplement: Supplementary file 2 — Fig. S2 The expression of “readers” and “writers” in inflammatory macrophages. a-f Expression of the Mettl3 (a), Mettl14 (b), Mettl16 (c), WTAP (d), ALKBH5 (e) and FTO (f) mRNAs was measured in THP-1 cells stimulated with TNF-α (10 ng/ml) using qRT-PCR. n=3 samples per group. The data are presented as the means ± SD. P-values were determined using Student’s t-test. *, P<0.05; ****, P<0.0001; ns, not significant. g-l Expression of the Mettl3 (g), Mettl14 (h), Mettl16 (i), WTAP (j), ALKBH5 (k) and FTO (l) mRNAs was measured in peritoneal macrophages from the mouse endotoxemia model using qRT-PCR. n=4 samples per group. The data are presented as the means ± SD. P-values were determined using Student’s t-test. *, P<0.05; **, P<0.01; ***, P<0.001; ns, not significant (PDF 926 KB) [file 18_2022_4331_MOESM2_ESM.pdf]

**a**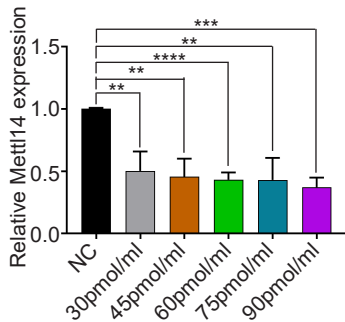**b**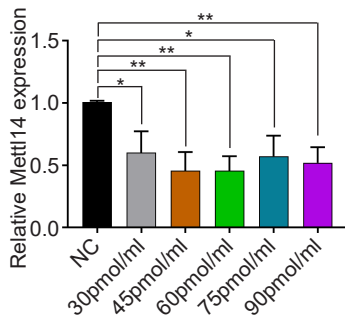**c**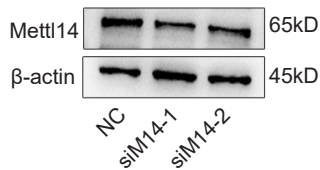**d**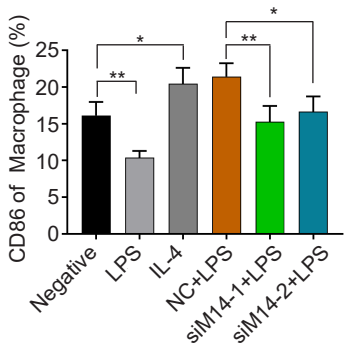**e**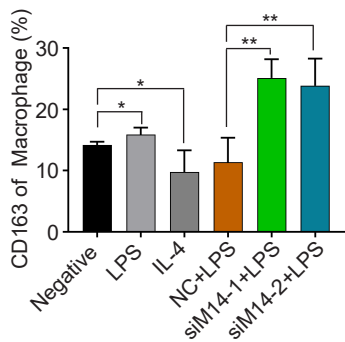

Supplement: Supplementary file 3 — Fig. S3 Mettl14 knockdown promotes the M2 polarization of macrophages. a-b Expression of the Mettl14 mRNA was measured in THP-1 cells transfected with siMettl14 using qRT-PCR. n=3 per group. The data are expressed as the mean ± SD. P-values were determined by one-way ANOVA with Fisher’s LSD post-hoc test. *, P<0.05; **, P<0.01; ***, P; ****, P<0.0001. c Mettl14 protein level in THP-1 cells transfected with siMettl14. d-e THP-1 cells treated with NC, siM14-1, or siM14-2 before treatment with LPS (500 ng/ml) or IL-4 (20 ng/ml). Percentages of macrophages exhibiting M1 (CD68+CD86+) and M2 (CD68+CD163+) polarization were determined using flow cytometry. n=5 per group. The data are expressed as the mean ± SD. P-values were determined by one-way ANOVA with Fisher’s LSD post-hoc test. *, P<0.05; **, P<0.01 (PDF 909 KB) [file 18_2022_4331_MOESM3_ESM.pdf]

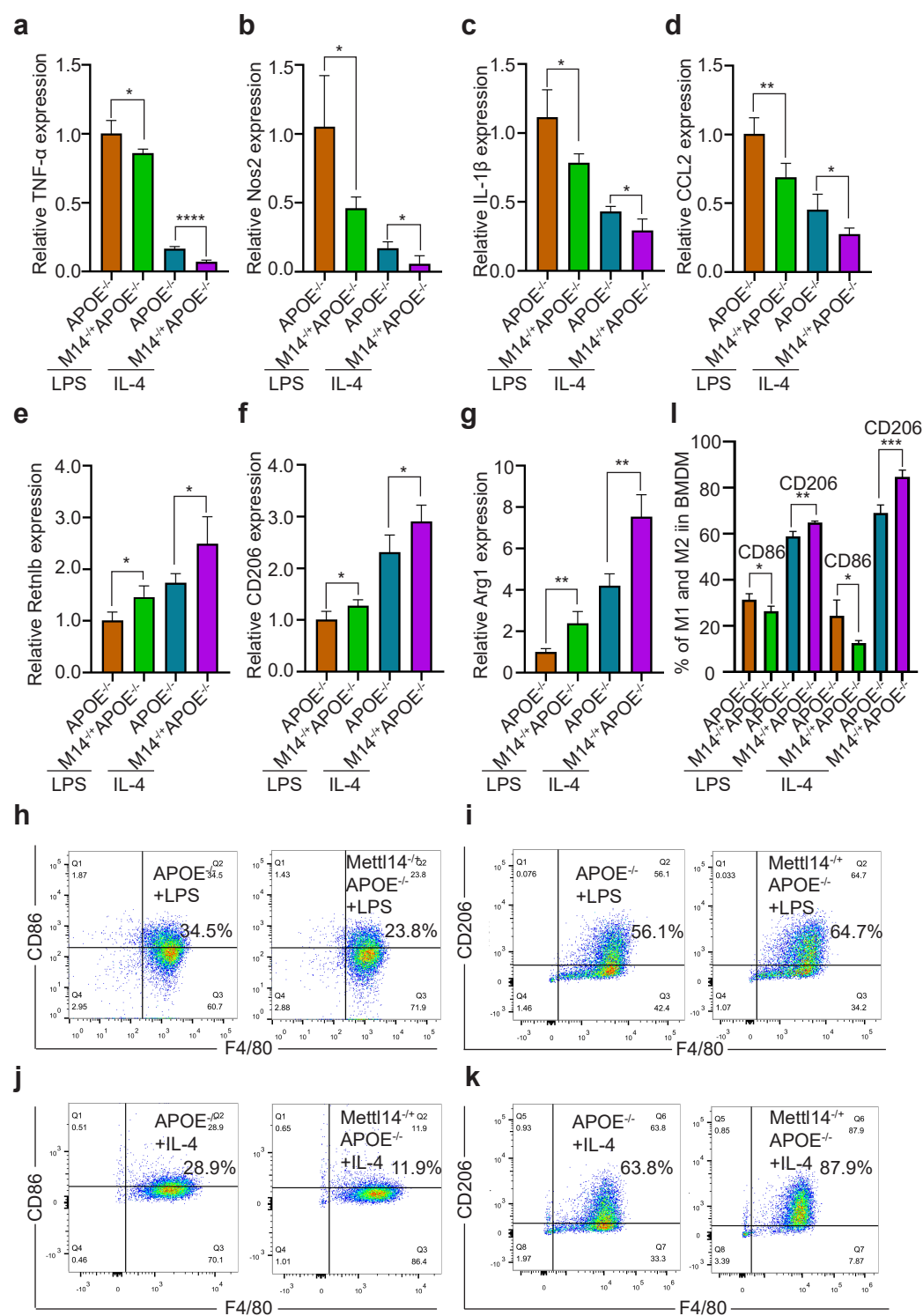

Supplement: Supplementary file 4 — Fig. S4 Mettl14 knockdown promotes M2 polarization of BMDMs. The BMDMs were stimulated with LPS (200 ng/ml) or IL-4 (20 ng/ml) on day 7 and harvested 24 h later. a-g TNF-α (a), Nos2 (b), IL-1β(c), CCL2 (d), Retnlb (e), CD206 (f), and Arg1 (g) mRNA levels in WT and KO mice. n=4 mice per group. The data are presented as the means ± SD. P-values were determined using Student’s t-test. *, P<0.05; **, P<0.01; ****, P<0.0001. h-l Percentages of macrophages exhibiting M1 (F4/80+CD86+) and M2 (F4/80+CD206+) polarization were determined using flow cytometry. n=4 samples per group. The data are presented as the means ± SD. P-values were determined using Student’s t-test. *, P<0.05; **, P<0.01; ***, P<0.001 (PDF 1728 KB) [file 18_2022_4331_MOESM4_ESM.pdf]

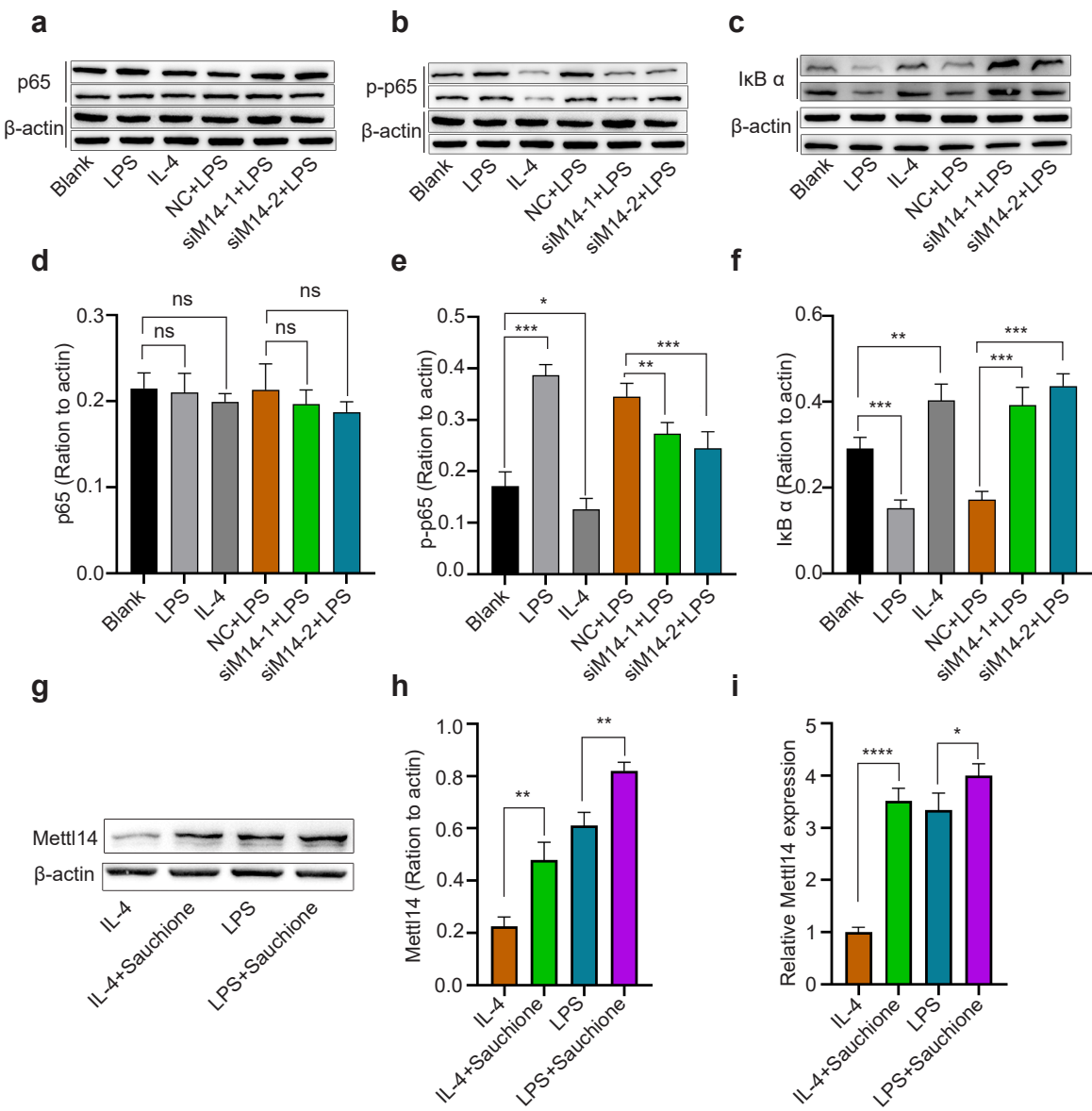

Supplement: Supplementary file 5 — Fig. S5 Mettl14 knockdown promotes the M2 polarization of macrophages. a-f Protein levels of p65 (a, d), p-p65 (b, e), and IκB-α (c, f) were determined using western blotting. n=3 per group. The data are expressed as the mean ± SD. P-values were determined by one-way ANOVA with Fisher’s LSD post-hoc test. *, P<0.05; **, P<0.01; ***, P<0.001; ns, not significant. g-h Mettl114 protein levels were measured using western blotting. The data are presented as the means ± SD. P-values were determined using Student’s t-test. **, P<0.01. i Mettl14 mRNA expression. The data are presented as the means ± SD. P-values were determined using Student’s t-test. *, P<0.05; ****, P<0.0001 (PDF 1098 KB) [file 18_2022_4331_MOESM5_ESM.pdf]

**a**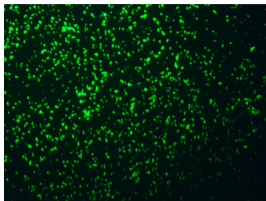

Blank

**b**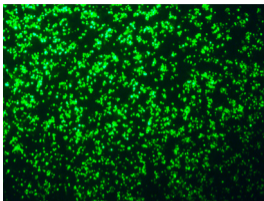

LPS

**c**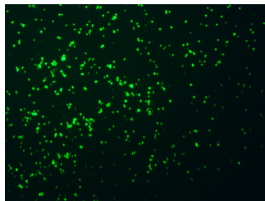

IL-4

**d**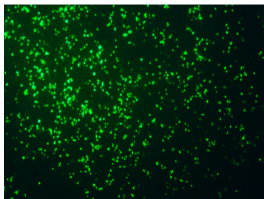

NC+LPS

**e**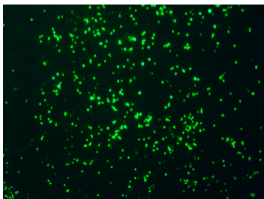

siM14-1+LPS

**f**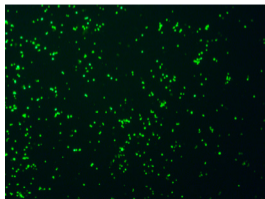

siM14-2+LPS

Supplement: Supplementary file 6 — Fig. S6 Mettl14 knockdown inhibited the adhesion of macrophages. a-f THP-1 cells were transfected with NC, OE Mettl14 or OE Mettl14 and siMyd88 before treatment with LPS (500 ng/ml). Representative fluorescence microscopy images of macrophages adhering to endothelial cells are shown (PDF 1761 KB) [file 18_2022_4331_MOESM6_ESM.pdf]

**a**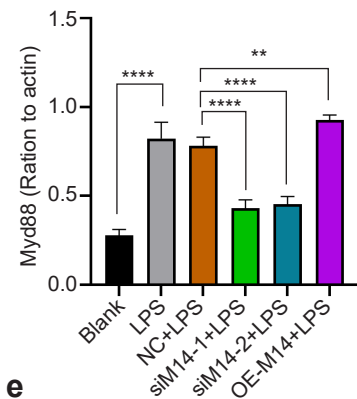**b**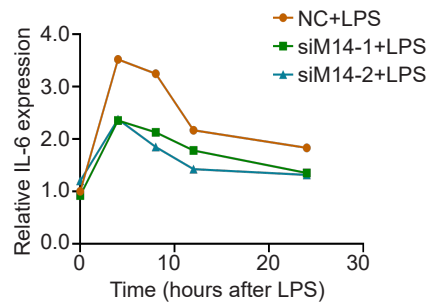**c**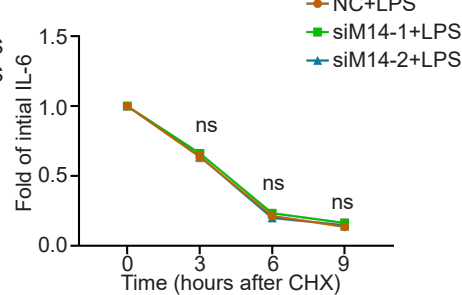**d**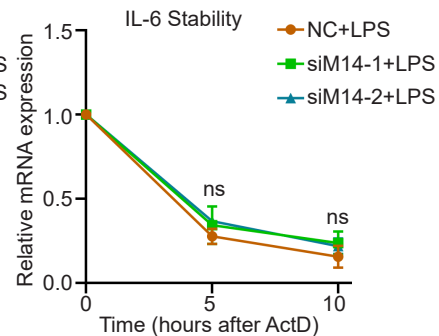**e**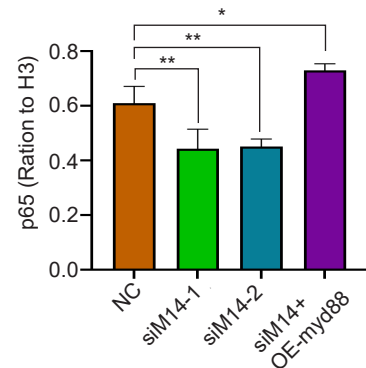**f**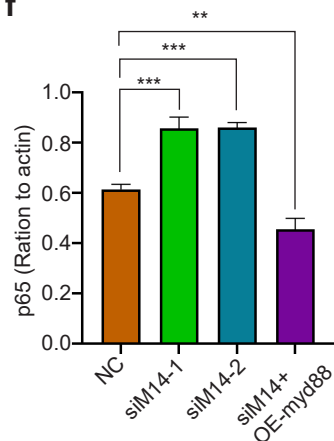**g**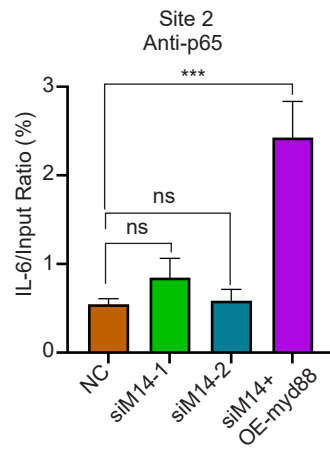

+LPS

Supplement: Supplementary file 7 — Fig. S7 Mettl14 regulates the expression of Myd88 and IL-6. a Myd88 protein levels were determined by western blotting. n=3 per group. The data are expressed as the mean ± SD. P-values were determined by one-way ANOVA with Fisher’s LSD post-hoc test. **, P<0.01; ****, P<0.0001. b IL-6 expression. c At 24 h after transfection, THP-1 cells were treated with cycloheximide (CHX). IL-6 protein levels were determined by western blotting. n=3 per group. The data are expressed as the mean ± SD. P-values were determined by one-way ANOVA with Fisher’s LSD post-hoc test. ns, not significant. d Results of the qRT-PCR-based analysis of IL-6 mRNA stability following treatment with actinomycin D (ActD, 2 μM). n=3 samples per group. The data are presented as the means ± SDs. P-values were determined using one-way ANOVA with Fisher’s LSD post-hoc test. ns, not significant. e-f The nuclear translocation of p65 was analyzed, and its levels in the cytosol and nucleus were quantified separately using western blotting. n=3 per group. The data are presented as the means ± SDs. P-values were determined using one-way ANOVA with Fisher’s LSD post-hoc test. *, P<0.05; **, P<0.01; ***, P<0.001. g Results of the ChIP assay showing the ability of the p65 protein to bind to IL-6 promoters. n=3 per group. The data are expressed as the mean ± SD. P-values were determined by one-way ANOVA with Fisher’s LSD post-hoc test. ***, P<0.001; ns, not significant (PDF 979 KB) [file 18_2022_4331_MOESM7_ESM.pdf]

**a**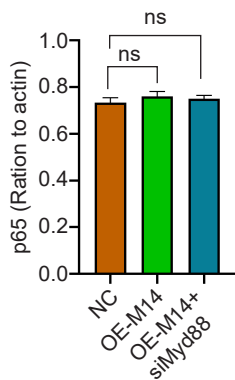**b**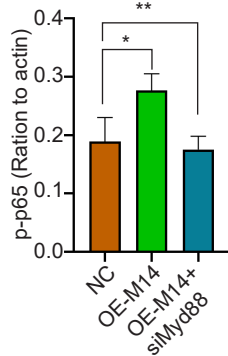**c**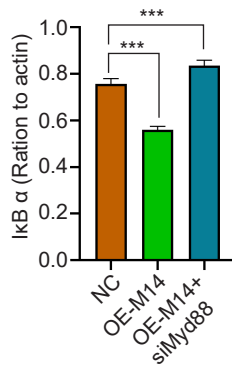**d**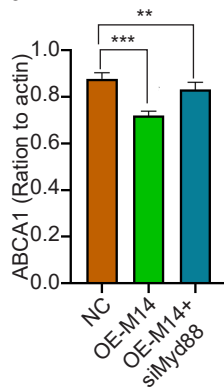**+LPS****e**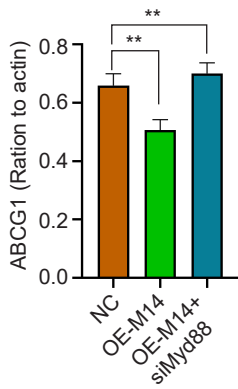**f**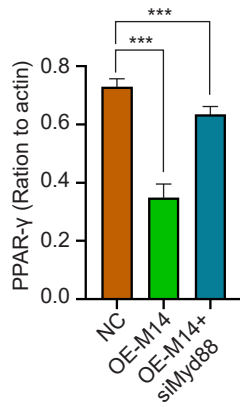**g**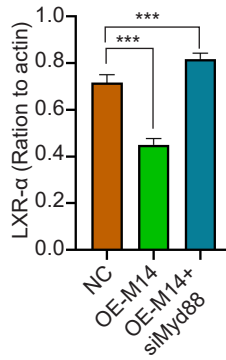**+LDL**

Supplement: Supplementary file 9 — Fig. S9 Mettl14 regulates the inflammatory response of macrophages through Myd88. a-c THP-1 cells were transfected with NC, OE Mettl14 or OE Mettl14 and siMyd88 before treatment with LPS (500 ng/ml). d-g THP-1 cells were transfected with NC, siM14-1, or siM14-2 before treatment with LDL (50 μg/ml). Protein levels of p65 (a), p-p65 (b), IκBα (c), ABCA1 (d), ABCG1 (e), PPAR-γ (f) and LXR-α (g) were measured by western blotting. n=3 per group. The data are expressed as the mean ± SD. P-values were determined by one-way ANOVA with Fisher’s LSD post-hoc test. *, P<0.05; **, P<0.01; ***, P<0.001; ns, not significant (PDF 943 KB) [file 18_2022_4331_MOESM9_ESM.pdf]

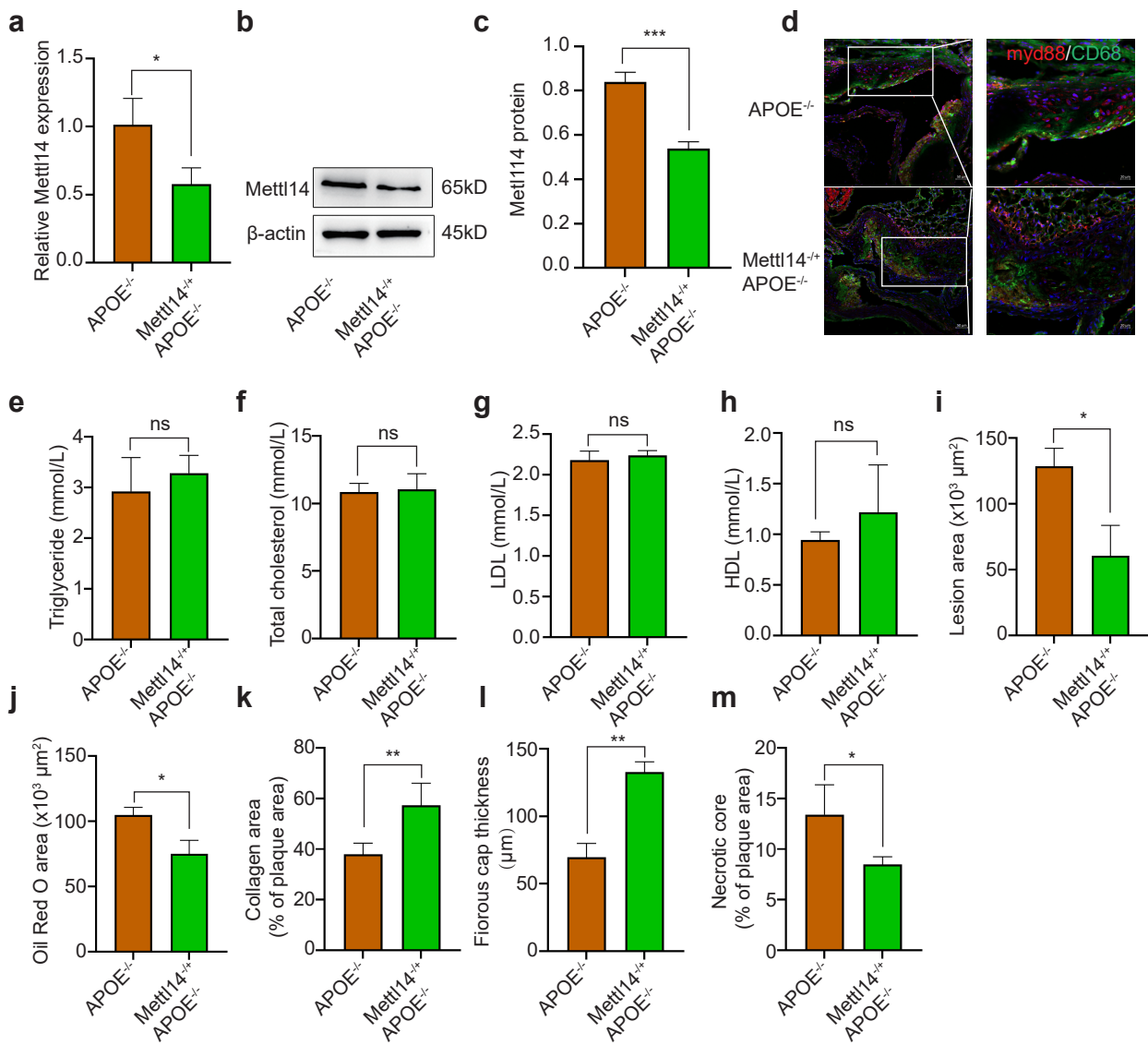

Supplement: Supplementary file 10 — Fig. S10 The development of atherosclerosis is inhibited in Mettl14 knockdown mice. a-c BMDMs were directly harvested on day 7 without stimulation. Mettl14 expression was detected in WT and KO mice using qRT-PCR and western blotting. n=3 animals per group. The data are presented as the means ± SD. P-values were determined using Student’s t-test. *, P<0.05; ***, P<0.001. Levels of triglycerides (e), total cholesterol (f), LDL (g) and HDL (h) in WT and KO mice. n=3 per group. The data are expressed as the mean ± SD. P-values were determined by Student’s t-test. ns, not significant. Quantification of the aortic root lesion area in sections stained with HE (i) and Oil red O (j), along with the collagen area (k), fibrous cap thickness (l) and necrotic core (m). n=3 per group. The data are expressed as the mean ± SD. P-values were determined by Student’s t-test. *, P<0.05; **, P<0.01 (PDF 2858 KB) [file 18_2022_4331_MOESM10_ESM.pdf]
