# Supplementary material for: Mettl14 mediates the inflammatory response of macrophages in atherosclerosis through the NF-κB/IL-6 signaling pathway
Source: Cell Mol Life Sci. 2022 May 22;79(6):311. doi: 10.1007/s00018-022-04331-0 (PMC9124663; doi:10.1007/s00018-022-04331-0)
Supplement: Supplementary file 8 — Fig. S8 An analysis of the Myd88 3’-UTR sequence revealed four matches to the 5’-RRACU-3’ m6A consensus sequence (R=A or G) (PDF 144 KB) [file 18_2022_4331_MOESM8_ESM.pdf]

1 AAUGCGACCCGACCGCGCUGAGGCUCCAGGACCGCCCGCCUUGGCUGCAG 50
51 GAGGUCCCGGCGCGGGUCUGCGGCCCCGGUCUCCUCCACAUCCUCCCUU 100
101 CCCCUGGCUGCUCUCAACAUGCGAGUGCGGCGCCGCCUGUCUCUGUUCUU 150
151 GAACGUGCGGACACAGGUGGCGGCCGACUGGACCGCGCUGGCGGAGGAGA 200
201 UGGACUUUGAGUACUUGGAGAUCCGGCAACUGGAGACACAAGCGGACCCC 250
251 ACUGGCAGGCUGCUGGACGCCUGGCAGGGACGCCCUGGCGCCUCUGUAGG 300
301 CCGACUGCUCGAGCUGCUUACCAAGCUGGGCCGCGACGACGUGCUGCUGG 350
351 AGCUGGGACCCAGCAUUGGGCAUAUGCCUGAGCGUUUCGAUGCCUUCAUC 400
401 UGCUAUUGCCCCAGCGACAUCCAGUUUGUGCAGGAGAUGAUCCGGCAACU 450
451 GGAACAGACAAACUAUCGACUGAAGUUGUGUGUGUCUGACCGCGAUGUCC 500
501 UGCCUGGCACCUGUGUCUGGUCUAUUGCUAGUGAGCUCAUCGAAAAGAGG 550
551 UGCCGCCGGAUGGUGGUGGUUGUCUCUGAUGAUUACCUGCAGAGCAAGGA 600
601 AUGUGACUUCCAGACCAAAUUUGCACUCAGCCUCUCUCCAGGUGCCCAUC 650
651 AGAAGCGACUGAUCCCCAUCAAGUACAAGGCAAUGAAGAAAGAGUUCCCC 700
701 AGCAUCCUGAGGUUCAUCACUGUCUGCGACUACACCAACCCCUGCACCAA 750
751 AUCUUGGUUCUGGACUCGCCUUGCCAAGGCCUUGUCCCUGCCCUGAAGAC 800
801 UGUUCUGAGGCCCUGGGUGUGUGUGUAUCUGUCUGCCUGUCCAUGUACUU 850
851 CUGCCCUGCCUCCUCCUUUCGUUGUAGGAGGAAUCUGUGCUCUACUUACC 900
901 UCUCAAUUCCUGGAGAUGCCAACUUCACAGACACGUCUGCAGCAGCUGGA 950
951 CAUCACAUUUCAUGUCCUGCAUGGAACCAGUGGCUGUGAGUGGCAUGUCC 1000
1001 ACUUGCUGGAUUAUCAGCCAGGACACUAUAGAACAGGACCAGCUGAGACU 1050
1051 AAGAAGGACCAGCAGAGCCAGCUCAGCUCUGAGCCAUUCACACAUCUUCA 1100
1101 CCCUCAGUUUCCUCACUUGAGGAGUGGGAUGGGGAGAACAGAGAGUAGCU 1150
1151 GUGUUUGAAUCCCUGUAGGAAAUGGUGAAGCAUAGCUCUGGGUCUCCUGG 1200
1201 GGGAGACCAGGCUUGGCUGCGGGAGAGCUGGCUGUUGCUGGACUACAUGC 1250
1251 UGGCCACUGCUGUGACCACGACACUGCUGGGGCAGCUUCUUCCACAGUGA 1300
1301 UGCCUACUGAUGCUUCAGUGCCUCUGCACACCGCCCAUUCCACUUCCUCC 1350
1351 UUCCCCACAGGGCAGGUGGGGAAGCAGUUUGGCCCAGCCCAAGGAGACCC 1400
1401 CACCUUGAGCCUUAUUUCCUAAUGGGUCCACCUCUCAUCUGCAUCUUUCA 1450
1451 CACCUCCCAGCUUCUGCCCAACCUUCAGCAGUGACAAGUCCCCAAGAGAC 1500
1501 UCGCCUGAGCAGCUUGGGCUGCUUUUCAUUUCCACCUGUCAGGAUGCCUG 1550
1551 UGGUCAUGCUCUCAGCUCCACCUGGCAUGAGAAGGGAUCCUGGCCUCUGG 1600
1601 CAUAUUCAUCAAGUAUGAGUUCUGGGGAUGAGUCACUGUAAUGAUGUGAG 1650
1651 CAGGGAGCCUUCCUCCCUGGGCCACCUGCAGAGAGCUUUCCCACCAACUU 1700
1701 UGUACCUUGAUUGCCUUACAAAGUUAUUUGUUUACAAACAGCGACCAUAU 1750
1751 AAAAGCCUCCUGCCCCAAAGCUUGUGGGCACAUGGGCACAUACAGACUCA 1800
1801 CAUACAGACACACACAUAUAUGUACAGACAUGUACUCUCACACACACAGG 1850
1851 CACCAGCAUACACACGUUUUUCUAGGUACAGCUCCCAGGAACAGCUAGGU 1900
