# Supplementary material for: Mettl14 mediates the inflammatory response of macrophages in atherosclerosis through the NF-κB/IL-6 signaling pathway
Source: Cell Mol Life Sci. 2022 May 22;79(6):311. doi: 10.1007/s00018-022-04331-0 (PMC9124663; doi:10.1007/s00018-022-04331-0)
Supplement: Supplementary file 11 — Supplementary file11 (DOC 57 KB) [file 18_2022_4331_MOESM11_ESM.doc]

**Table S1. The list of PCR primer sequences** and siRNA sequences

| Primer name | Sequence 5’---3’ |
| --- | --- |
| Mettl3 FO | TTGTCTCCAACCTTCCGTAGT |
| Mettl3 RE | CCAGATCAGAGAGGTGGTGTAG |
| Mettl14 FO | AGTGCCGACAGCATTGGTG |
| Mettl14 RE | GGAGCAGAGGTATCATAGGAAGC |
| Mettl16 FO | TTCTGTCAAGGTCGGACAATG |
| Mettl16 RE | CAGCACCACGAATGTTATGGG |
| WTAP FO | CTTCCCAAGAAGGTTCGATTGA |
| WTAP RE | TCAGACTCTCTTAGGCCAGTTAC |
| FTO FO | AACACCAGGCTCTTTACGGTC |
| FTO RE | TGTCCGTTGTAGGATGAACCC |
| ALKBH5 FO | CGGCGAAGGCTACACTTACG |
| ALKBH5 RE | CCACCAGCTTTTGGATCACCA |
| IL-1β FO | TGGCTTATTACAGTGGCAATGAG |
| IL-1β RE | GTAGTGGTGGTCGGAGATTCG |
| TNF-α FO | GTCTGGGCAGGTCTACTTTGG |
| TNF-α RE | GAGGTTGAGGGTGTCTGAAGG |
| IL-10 FO | GTTGTTAAAGGAGTCCTTGCTG |
| IL-10 RE | TTCACAGGGAAGAAATCGATGA |
| CD163 FO | CATTATGTCCTTCAGAGCAAGTG |
| CD163 RE | AGCGACCTCCTCCATTTACC |
| Myd88 FO | GGCTGCTCTCAACATGCGA |
| Myd88 RE | CTGTGTCCGCACGTTCAAGA |
| IL-6 FO | AGCCACTCACCTCTTCAGAAC |
| IL-6 RE | GCAAGTCTCCTCATTGAATCCAG |
| β-actin FO | TCATGAAGTGTGTGACGTGGACATC |
| β-actin RE | CAGCAGGAGCAATGATCTTGATCT |
| ICAM-1 FO | TTGGGCATAGAGACCCCGTT |
| ICAM-1 RE | GCACATTGCTCAGTTCATACACC |
| Myd88 RIP site 1 FO | GCAGCAGCTGGACATCACAT |
| Myd88 RIP site 1 RE | CTGGCTCTGCTGGTCCTTCT |
| Myd88 RIP site 2 FO | TCCTCCACATCCTCCCTTCC |
| Myd88 RIP site 2 RE | CAGTTGCCGGATCTCCAAGT |
| Myd88 RIP site 3 FO | CTGGCTGCTCTCAACATGCG |
| Myd88 RIP site 3 RE | CCGCTTGTGTCTCCAGTTGC |
| Myd88 RIP site 4 FO | ATGGTGAAGCATAGCTCTGGG |
| Myd88 RIP site 4 RE | ACTGTGGAAGAAGCTGCCC |
| IL-6 CHIP site 1 FO | ACGTTTTTGGAGCAAGGTAGAG |
| IL-6 CHIP site 1 RE | TAGGGAGCTTGGAGACACTG |
| IL-6 CHIP site 2 FO | AGGAGTTCAAGACCCGCCTG |
| IL-6 CHIP site 2 RE | GACTACAGATGCATGCGACCAC |
| siMettl14-1 sense | GCAGCACCUCGAUCAUUUATT |
| siMettl14-1 antisense | UAAAUGAUCGAGGUGCUGCTT |
| siMettl14-2 sense | CCUGGGAAGACUAAGACUUTT |
| siMettl14-2 antisense | AAGUCUUAGUCUUCCCAGGTT |
| siMyd88 sense | CCGGCAACUGGAGACACAATT |
| siMyd88 antisense | UUGUGUCUCCAGUUGCCGGTT |
| Negative Control sense | UUCUCCGAACGUGUCACGUTT |
| Negative Control antisense | ACGUGACACGUUCGGAGAATT |
